# The effectiveness of peer support for individuals with mental illness: systematic review and meta-analysis

**DOI:** 10.1017/S0033291722002422

**Published:** 2023-08

**Authors:** Dorien Smit, Clara Miguel, Janna N. Vrijsen, Bart Groeneweg, Jan Spijker, Pim Cuijpers

**Affiliations:** 1 Pro Persona Mental Health Care, Pro Persona Research, Depression Expertise Center, Nijmegen, The Netherlands; 2 Behavioural Science Institute, Radboud University Nijmegen, Nijmegen, The Netherlands; 3 Department of Clinical, Neuro, and Developmental Psychology, Amsterdam Public Health Research Institute, Vrije Universiteit Amsterdam, Amsterdam, the Netherlands; 4 Department of Psychiatry, Donders Institute for Brain, Cognition and Behaviour, Radboud University Medical Center, Nijmegen, The Netherlands; 5 Dutch Depression (Patient) Association, Amersfoort, The Netherlands

**Keywords:** Clinical recovery, depression, functional recovery, mental illness, meta-analysis, peer support interventions, personal recovery, serious mental illness, systematic review

## Abstract

**Background:**

The benefits of peer support interventions (PSIs) for individuals with mental illness are not well known. The aim of this systematic review and meta-analysis was to assess the effectiveness of PSIs for individuals with mental illness for clinical, personal, and functional recovery outcomes.

**Methods:**

Searches were conducted in PubMed, Embase, and PsycINFO (December 18, 2020). Included were randomized controlled trials (RCTs) comparing peer-delivered PSIs to control conditions. The quality of records was assessed using the Cochrane Collaboration Risk of Bias tool. Data were pooled for each outcome, using random-effects models.

**Results:**

After screening 3455 records, 30 RCTs were included in the systematic review and 28 were meta-analyzed (4152 individuals). Compared to control conditions, peer support was associated with small but significant post-test effect sizes for *clinical recovery*, *g* = 0.19, 95% CI (0.11–0.27), *I*^2^ = 10%, 95% CI (0–44), and *personal recovery, g* = 0.15, 95% CI (0.04–0.27), *I^2^* = 43%, 95% CI (1–67), but not for *functional recovery*, *g* = 0.08, 95% CI (−0.02 to 0.18), *I^2^* = 36%, 95% CI (0–61). Our findings should be considered with caution due to the modest quality of the included studies.

**Conclusions:**

PSIs may be effective for the clinical and personal recovery of mental illness. Effects are modest, though consistent, suggesting potential efficacy for PSI across a wide range of mental disorders and intervention types.

## Introduction

In recent years mental health care services and social organizations increased their focus on implementing peer support initiatives to promote recovery and expand the availability of support for individuals coping with mental illness (Stratford et al., 2017). This growing interest in peer support is stimulated by the World Health Organization (WHO), as they consider it a feasible tool which adds a person-centered, recovery, and rights-based approach to biomedical practices in mental health services (WHO, 2021). Also, the (coronavirus disease 2019) COVID-19 pandemic increases the need for community-based interventions such as peer support (Suresh, Alam, & Karkossa, 2021), since mental health problems may have exacerbated and mental health services may be less accessible (Salari et al., 2020).

Peer support involves a mutual exchange of practical and emotional support, based on ‘shared understanding, respect, and mutual empowerment between people in similar situations’ (Mead, Hilton, & Curtis, 2001) with critical ingredients such as shared responsibility (Mead, 2003; Mead & MacNeil, 2006), hope, self-determination over one's life, and the use of lived experience knowledge (Repper & Carter, 2011; Slade et al., 2014; Solomon, 2004). These aspects are embedded within the varying peer support programs implementing different structures, content, duration, and delivery formats, targeting different populations, and evaluating a wide range of outcomes (Chien, Clifton, Zhao, & Lui, 2019; Lloyd-Evans et al., 2014).

Previous meta-analyses examining the effects of peer support interventions (PSIs) were focused on specific target groups, such as patients with (perinatal) (Huang et al., 2020) depression (Bryan & Arkowitz, 2015; Pfeiffer, Heisler, Piette, Rogers, & Valenstein, 2011) or serious mental illness (SMI) (Chien et al., 2019; Fuhr et al., 2014; Lloyd-Evans et al., 2014), or only analyze specific outcomes (e.g. cost-effectiveness; Chien et al., 2019; Huang et al., 2020) and empowerment (Burke, Pyle, Machin, Varese, & Morrison, 2019) or included either one-to-one (White et al., 2020) or group interventions (Lyons, Cooper, & Lloyd-Evans, 2021).

To the best of our knowledge, no previous meta-analysis has examined the effects of peer support across all patient groups and intervention types. We conducted a comprehensive systematic review and meta-analysis of randomized controlled trials (RCTs) comparing the effects of any peer support intervention with control conditions. We focused on 3 pre-specified main outcomes – clinical, personal, and functional recovery – and, when possible, we also examined specific outcomes within these main categories (e.g. depressive symptoms, empowerment, and quality of life).

## Methods

### Protocol registration

This study adheres to the Preferred Reporting Items for Systematic Reviews and Meta-analyses (PRISMA) reporting guideline (Moher, Liberati, Tetzlaff, Altman, & The, 2009), and focuses on the effect of peer support for individuals with mental health disorders, corresponding to the main part of our protocol (https://osf.io/58urb). This protocol also includes our search for RCTs on peer support for relatives and caregivers of individuals with mental illness, which will not be reported here.

### Search strategy

We searched PubMed, Embase, and PsycINFO up to December 18^th^ 2020, without language restriction. We used index terms from database-specific thesauruses as well as free text words indicative of mental illness and peer support (search strings are available in Appendix A). References of included trials and previous systematic reviews were reviewed for eligibility.

### Identification and selection of studies

Two authors (DS and CM) independently screened titles and abstracts to identify eligible papers for inclusion. To determine final inclusions, full texts of the selected papers were examined. We included studies: (a) that were RCTs; (b) comparing any PSI format; (c) for adults with a clinical or self-reported mental disorder diagnosis, or a score above a cut-off on a standardized mental disorder symptom measure; (d) with care-as-usual (CAU), waiting list (WL), or other active (e.g. clinician-led therapies) or inactive comparators (e.g. an attention control website) (Griffiths et al., 2012); and (e) outcomes focusing on at least one of 3 categories: *clinical* (i.e. symptomatic) recovery (Slade et al., 2014; van Eck, Burger, Vellinga, Schirmbeck, & de Haan, 2018); *personal* recovery (e.g. empowerment; Mueser et al., 2006; van Weeghel, van Zelst, Boertien, & Hasson-Ohayon, 2019); *functional* recovery (e.g. quality of life; Mueser et al., 2006). For a definition of the categories, see Appendix B. Peers are defined as individuals recovered or in recovery from a mental illness. We excluded trials when the intervention was partially or co-delivered by a non-peer (e.g. a lay health worker), targeting substance use, somatic disorder self-management, or including (ex-)employees with mental illness due to their job (e.g. veterans). Any disagreement was resolved with a third author (PC), and central issues were discussed in meetings with all authors.

### Data extraction and risk of bias assessment

A standardized form was used by 2 authors (DS and CM) to extract data regarding study context, participants' and intervention characteristics, including diagnoses, intervention format, control condition, and outcome data. When multiple measurements or control groups were available, we followed our developed decision tool (see Appendix C).

Study authors DS and CM independently assessed included trials using the Cochrane Collaboration Risk of Bias (RoB) tool 2.0 (Higgins et al., 2011), resolving any discrepancy with a third researcher (PC). Each of the following RoB-domains was rated as high risk, some concerns, or low risk: (a) the randomization process; (b) deviations from the intended interventions; (c) missing outcome data (up to 10% drop out was rated as low risk); (d) inappropriate measurement of the outcome; (e) selection of the reported result. An overall RoB score was calculated for each study, following our approach as presented in Appendix C.

### Outcome measures

Outcomes included three pre-specified recovery categories: (1) *clinical recovery*, indicating the degree of psychiatric symptomatology (Slade et al., 2014), with measures including the Brief Symptom Inventory (BSI), and Brief Psychiatric Rating Scale (BPRS); (2) *personal recovery*, concerning the extents of perceived recovery, sense of purpose, and personal agency [Mueser et al., 2006; e.g., Recovery Assessment Schedule (RAS), Empowerment Scale (ES)]; (3) *functional recovery*, referring to the quality of life and the degree of vocational and social functioning [Robinson, Woerner, McMeniman, Mendelowitz, & Bilder, 2004; e.g., World Health Organization Quality of Life (WHOQOL), EuroQoL 5D (EQ-5D)].

Also, we examined subcategories within the main categories of outcomes: *clinical* recovery (*depressive symptoms*), *personal* recovery (*empowerment, RAS, hope*), and *functional* recovery (*quality of life, social support*, and *loneliness*). These subcategories of specific outcomes were pooled when a minimum of five trials were available. In Appendix B, a comprehensive definition for each outcome category is provided, with details on data extraction per category described in Appendix C, and corresponding instruments in Appendix D.

### Statistical analysis

We conducted separate meta-analyses comparing PSIs and control conditions for each main group of outcomes (clinical, functional, and personal recovery) as well as subcategories of outcomes within the main groups (e.g. hope, quality of life). Effects were estimated at post-test, and when possible, at long-term follow-ups (⩾6 months after randomization).

We calculated between-group effect sizes (Hedges' *g*) by using means, standard deviations and N. When these were not reported, we used dichotomous outcomes or other statistics (e.g. *p* value, *t* value) for calculating effect sizes. Intention-to-treat data were used. Effect sizes were pooled with a random-effects model, using the Hartung-Knapp-Sidik-Jonkman method (IntHout, Ioannidis, & Borm, 2014). Heterogeneity was estimated with the *I*^2^ statistic and its 95% confidence interval (CI). In addition, we included prediction intervals (PI), which represent 95% CI of the predictive distribution of effects in future comparable trials.

Categorical moderators of effects were explored in subgroup analyses by using a mixed-effects model. We conducted subgroup analyses when a minimum of three studies were available per subgroup.

We estimated publication bias through visual funnel plot inspection, Egger's test (Egger, Smith, Schneider, & Minder, 1997), and with Duval and Tweedie trim-and-fill procedure (Duval & Tweedie, 2000). We conducted sensitivity analyses by: (a) excluding outliers (defined as studies whose 95% CI effect size did not overlap with the 95% CI of the pooled effect), and (b) exploring the influence of RoB in the results.

All meta-analyses were conducted in version 4.1.1 of R, using the packages *meta* (Balduzzi, Rücker, & Schwarzer, 2019), *metafor* (Viechtbauer, 2010), and *dmetar* (Harrer, Cuijpers, Furukawa, & Ebert, 2019).

## Results

### Inclusion of studies

**Fig. 1.**
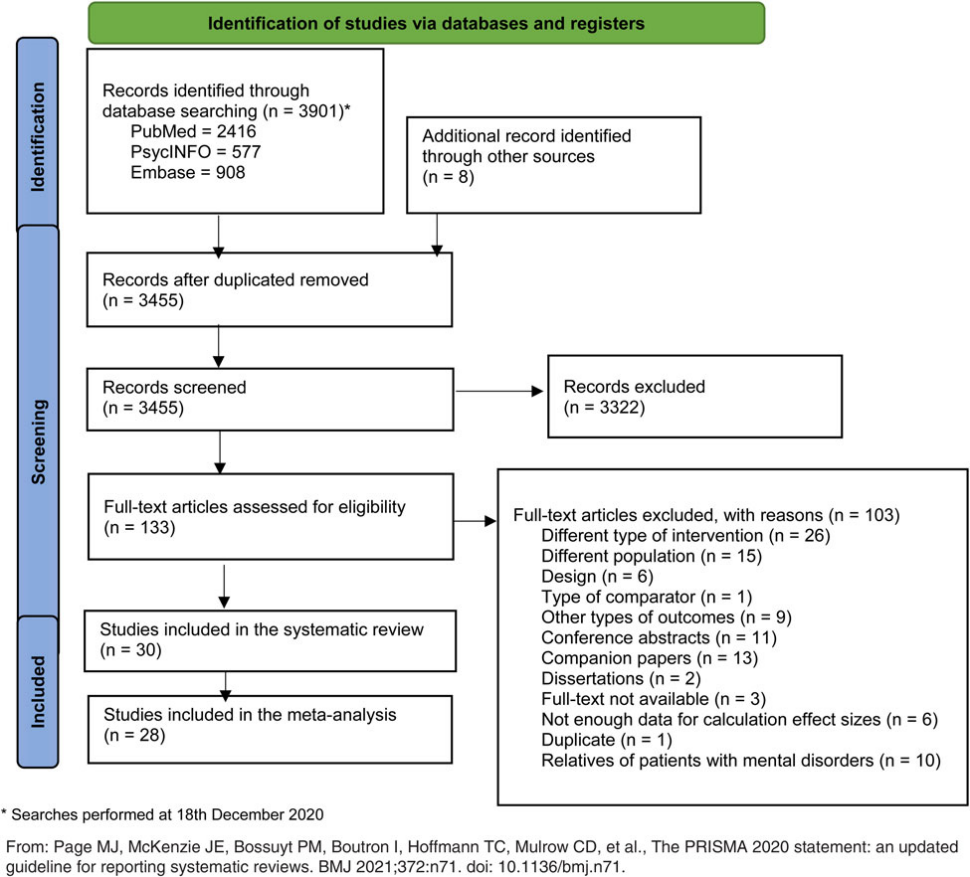
Flowchart of Selection and Inclusion Process, Following the PRISMA Statement

The PRISMA flowchart is presented in Fig. 1. We screened 3455 hits, and we examined the full-text of 133 studies. A total of 30 studies (for references, see Appendix E) were included, of which 28 trials and 4152 participants, were included in the meta-analysis. Three studies (Field, Diego, Delgado, & Medina, 2013; Ludman et al., 2007; Mathews et al., 2018) included a clinician-led group as comparator [e.g. Interpersonal Psychotherapy (IPT) or Cognitive Behavioral Therapy (CBT)], including one overlapping trial (Ludman et al., 2007) which examined a control condition and a clinician-led comparator. Due to the limited number of studies, we did not pool trials with clinician-led comparators. A narrative description of these studies is presented in Appendix F.

### Study characteristics

Selected characteristics of 30 included studies are presented in Appendix D. Two main subgroups were identified across the included trials: patients with SMI (20 trials) and individuals with depression (7 trials). SMI studies included a heterogeneous group of patients including but not limited to psychosis, depressive disorders, anxiety disorders, or bipolar disorders. The majority of depression studies (5 trials) focused on perinatal depression (Dennis, 2003; Dennis et al., 2009; Gjerdingen, McGovern, Pratt, Johnson, & Crow, 2013; Letourneau et al., 2011; Shorey et al., 2019), with participants scoring above a cut-off on a questionnaire. One study focused on women with eating disorders (Ranzenhofer et al., 2020). Most studies had CAU (16 trials) or WL (9 trials) as a control condition.

In 12 trials the PSI consisted of group meetings, 17 evaluated one-to-one peer support, and one trial implemented a mixed format. Face-to-face delivery was most common (16 trials), three trials evaluated telephone-based support, two trials examined internet support groups, and nine trials examined a mixed intervention, bringing together the latter formats. Intervention duration and frequency were heterogeneous and reported inconsistently, ranging from three weeks to six months with weekly meetings or a more flexible frequency.

### Risk of bias

Overall, there is a high RoB in the majority of included studies: 21 trials were rated at high risk (21/30, 70%), six studies were judged as having some concerns for risk of bias (6/30, 20%), and only three studies met criteria for low risk of bias (3/30, 10%). Focusing on the separate RoB domains, twelve studies (12/30, 40%) were rated at low risk of bias for domain 1, due to reporting an adequate randomization process. Due to the unstructured naturalistic approach of peer support, 23 studies (23/30, 77%) were rated at low risk in domain 2 (deviations from the intended interventions). Ten trials (10/30, 33%) were rated as low RoB in domain 3 due to missing outcome data. Thirteen trials (13/30, 43%) were judged at low risk in domain 4 due to measurement of the outcome, using self-report measures only. For domain 5, only five studies (5/30, 17%) were prospectively registered and were rated at low risk (see Figures G1 and G2 in Appendix G, and Appendix H for RoB rating per domain and study).

### Clinical recovery

**Fig. 2.**
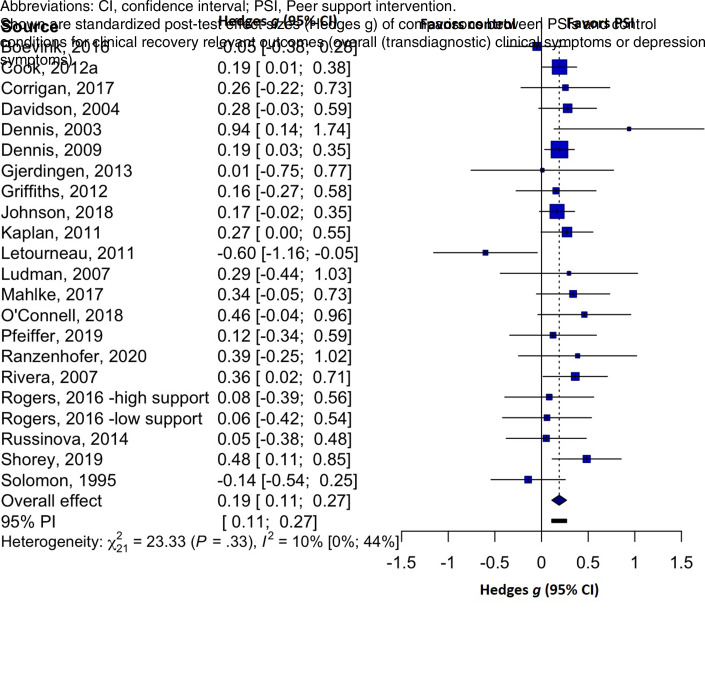
Effect sizes of clinical recovery outcomes.

The pooled effect size at post-test across 22 PSI studies measuring *clinical recovery* was significant, with *g* *=* 0.19, 95% CI (0.11–0.27) (see Table 1 and Fig. 2). Heterogeneity was low, *I*^2^ = 10%, 95% CI (0–44). The PI was consistent with benefit, overlapping completely with the 95% CI.

**Table 1.** Effects for clinical recovery of peer support interventions compared with CAU, WL or other control conditions: Hedges *g*^a^

Clinical recovery	No. of trials	*g* (95% CI)	*I*^2^ (95% CI)	*p*	PI
Main effect					
All studies pooled	22	0.19 (0.11–0.27)	10 (0–44)	<0.001	0.11–0.27
Subgroup of patients					
Individuals clinically diagnosed with SMI	14	0.18 (0.10–0.26)	0 (0–55)	<0.001	0.10–0.26
Individuals with depressive symptoms (*k* = 6 cut-off, *k* = 1 diagnosis)^b^	7	0.19 (−0.20 to 0.58)	57 (0–81)	0.27	−0.67 to 1.05
Specific outcomes					
Depressive symptoms	12	0.14 (−0.02 to 0.30)	41 (0–70)	0.09	−0.13 to 0.41
Publication bias					
Adjusting for publication bias^c^	23	0.18 (0.10–0.27)	18 (0–50)	<0.001	0.10–0.27
Sensitivity analyses					
Outlier excluded	21	0.20 (0.14–0.27)	0 (0–47)	<0.001	0.14–0.27
Risk of bias^d^					
High risk	15	0.15 (0.06–0.25)	11 (0–49)	0.001	NA
Some concerns	4	0.20 (0.14–0.27)	0 (0–85)	<0.001	NA
Low risk	3	0.52 (0.29–0.76)	0 (0–90)	<0.001	NA
Long-term					
6 to 9 months	13	0.17 (0.08–0.26)	0 (0–57)	0.002	0.08–0.26
12 to 18 months^e^	8	0.10 (−0.21 to 0.40)	63 (20–83)	0.48	−0.65 to 0.84

CAU, care-as-usual; CI, confidence interval; NA, not applicable; PI, prediction interval; WL, waiting list.

a According to the random-effects model.

b *k* = 6 studies included individuals with depressive symptoms scoring above a cut-off on a standardized mental disorder symptom measure (of which *k* = 5 are on perinatal depression), and *k* = 1 study included adults with a clinical diagnosis.

c Egger's test was not significant (*p* = 0.99) and the number of imputed studies using Duvall and Tweedie trim-and-fill procedure was 23.

d The *p* value for the between-group effect sizes is significant (*p* = 0.02).

e Of the *k* = 8 studies only one study included 18 months follow-up data, the remaining studies reported 12 months follow-up data.

For the subgroup of patients with SMI (Boevink, Kroon, van Vugt, Delespaul, & van Os, 2016; Cook et al., 2012a; Corrigan et al., 2017; Davidson et al., 2004; Johnson et al., 2018; Kaplan, Salzer, Solomon, Brusilovskiy, & Cousounis, 2011; Mahlke et al., 2017; O'Connell et al., 2018; Pfeiffer et al., 2019; Rivera, Sullivan, & Valenti, 2007; Rogers et al., 2016; Russinova et al., 2014; Solomon & Draine, 1995), the effect size was significant, *g* = 0.18, 95% CI (0.10–0.26) (14 trials). However, for the subgroup of patients with depression (Dennis, 2003; Dennis et al., 2009; Gjerdingen et al., 2013; Griffiths et al., 2012; Letourneau et al., 2011; Ludman et al., 2007; Shorey et al., 2019), no significant effects were detected, *g* *=* 0.19, 95% CI (−0.20 to 0.58) (7 trials). In the same line, no significant effects were found when pooling 12 trials that specifically reported *depression* outcomes. Subgroup analyses to examine potential moderators of intervention effects showed no significant differences between subgroups (see Appendix I). There were significant differences in effects based on RoB levels, *p* = 0.016; *Q*_2_ = 8.30, with the three studies rated at low risk showing a significant effect of *g* *=* 0.52, 95% CI (0.29–0.76).

Inspection of funnel plots, Egger's test, *p* = 0.99, and the trim-and-fill procedure did not indicate significant publication bias (see Figure J1 in Appendix J). Removing one outlier (Letourneau et al., 2011) did not have a substantial impact on the effect, *g* = 0.20, 95% CI (0.14–0.27).

Long-term effects for all clinical recovery outcomes indicated that the effect remained significant at six to nine months follow-up, *g* *=* 0.17, 95% CI (0.08–0.26), but not at 12 to 18 months follow-up, *g* *=* 0.10, 95% CI (−0.21 to 0.40).

### Personal recovery

The pooled effect size at post-test across 19 PSI studies measuring *personal recovery* was significant, *g* *=* 0.15, 95% CI (0.04–0.27) (see Table 2 and Figure K1 in Appendix K). Heterogeneity was moderate, *I^2^* = 43%, 95% CI (1–67), although the PI (−0.16–0.47) was wide and contained the null effect.

**Table 2.** Effects for personal recovery of peer support interventions compared with CAU, WL or other control conditions: Hedges *g*^a^

Personal recovery	No. of trials	*g* (95% CI)	*I*^2^ (95% CI)	*p*	PI
Main effect					
All studies pooled	19	0.15 (0.04–0.27)	43 (1–67)	0.01	−0.16 to 0.47
Subgroup of patients					
Individuals clinically diagnosed with SMI	17	0.15 (0.02–0.28)	48 (9–71)	0.02	−0.21 to 0.51
Individuals with depressive symptoms (both cut-off)^b^	2	0.18 (−1.11 to 1.46)	NA	0.33	NA
Specific outcomes					
Empowerment	13	0.25 (−0.10 to 0.60)	84 (74–90)	0.15	−0.97 to 1.47
Recovery (RAS)	8	0.21 (−0.05 to 0.47)	58 (9–81)	0.09	−0.39 to 0.81
Hope	5	0.13 (0.03–0.22)	0 (0–79)	0.02	0.02–0.23
Publication bias					
Adjusting for publication bias^c^	24	0.23 (0.12–0.35)	56 (31–72)	<0.001	−0.21 to 0.68
Sensitivity analyses					
Outlier excluded	18	0.13 (0.05–0.21)	1 (0–50)	0.003	−0.01 to 0.27
Risk of bias^d^					
High risk	14	0.15 (0.01–0.29)	56 (21–76)	0.003	NA
Some concerns	4	0.14 (0.03–0.24)	0 (0–85)	0.01	NA
Low risk	1	0.35 (−0.26 to 0.95)	NA (NA)	0.26	NA
Long-term					
6 to 9 months	12	0.10 (−0.10 to 0.30)	64 (32–81)	0.28	−0.48 to 0.68
12 to 18 months^e^	7	0.54 (−0.33 to 1.41)	93 (89–96)	0.18	−1.96 to 3.04

CAU, care-as-usual; CI, confidence interval; NA, not applicable; PI, prediction interval; WL, waiting list.

a According to the random-effects model.

b Both studies (*k* = 2) included individuals with perinatal depressive symptoms scoring above a cut-off on a standardized mental disorder symptom measure.

c Egger's test was not significant (*p* = 0.66) and the number of imputed studies using Duvall and Tweedie trim-and-fill procedure was 24.

d The *p* value for the between-group effect sizes is not significant (*p* = 0.79).

e Of the *k* = 7 studies, only one study included 18 months follow-up data, the remaining studies reported 12 months follow-up data.

For the subgroup of individuals with SMI (Boevink et al., 2016; Castelein et al., 2008; Cook et al., 2012a, 2012b; Corrigan et al., 2017, 2018; Davidson et al., 2004; Johnson et al., 2018; Kaplan et al., 2011; Mahlke et al., 2017; Pfeiffer et al., 2019; Rogers et al., 2016; Russinova et al., 2014; Rüsch et al., 2014; Salzer et al., 2016; van Gestel-Timmermans, Brouwers, van Assen, & van Nieuwenhuizen, 2012), the effect size was significant, *g* *=* 0.15, 95% CI (0.02–0.28) (17 trials). For individuals with depressive symptoms, the number of trials (Dennis, 2003; Griffiths et al., 2012) was too small to reliably detect effects. Pooling specific outcomes within personal recovery resulted in significant effects for *hope* outcomes, *g* *=* 0.13, 95% CI (0.03–0.22), but not for *empowerment* or the *Recovery Assessment Scale.* In subgroup analyses, we found no differences in the effect of PSIs among potential moderators (see Appendix I).

No indications of publication bias were observed, Egger's test, *p* = 0.66, see Figure J2 in Appendix J. The effect size did not substantially change when excluding one outlier (Salzer et al., 2016), *g* *=* 0.13, 95% CI (0.05–0.21). Subgroup analyses did not detect differences in effects between RoB levels, although only one trial was rated at low risk and the impact of RoB is uncertain due to lack of power.

Long-term effects for all personal recovery outcomes showed nonsignificant results for both periods, six to nine months: *g* *=* 0.10, 95% CI (−0.10 to 0.30), and 12 to 18 months follow-up: *g* *=* 0.54, 95% CI (−0.33 to 1.41).

### Functional recovery

The pooled effect size at post-test across 25 PSI studies measuring *functional recovery* was nonsignificant, *g* *=* 0.08, 95% CI (−0.02 to 0.18), with low heterogeneity, *I^2^* = 36%, 95% CI (0–61) (see Table 3 and Figure K2 in Appendix K). The PI was wide and contained the null effect (−0.16 to 0.32).

**Table 3.** Effects for functional recovery of peer support interventions compared with CAU, WL or other control conditions: Hedges *g*^a^

Functional recovery	No. of trials	*g* (95% CI)	*I*^2^ (95% CI)	*p*	PI
Main effect					
All studies pooled	25	0.08 (−0.02 to 0.18)	36 (0–61)	0.11	−0.16 to 0.32
Subgroup of patients					
Individuals clinically diagnosed with SMI	14	0.18 (0.10–0.26)	0 (0–55)	<0.001	0.10–0.26
Individuals with depressive symptoms (*k* = 6 cut-off, *k* = 1 diagnosis)^b^	6	0.02 (−0.34 to 0.37)	47 (0–79)	0.90	−0.66 to 0.70
Specific outcomes					
Quality of life	18	0.08 (−0.04 to 0.19)	32 (0–61)	0.18	−0.15 to 0.30
Social support	15	0.07 (−0.05 to 0.18)	13 (0–51)	0.25	−0.07 to 0.20
Loneliness	7	0.09 (−0.05 to 0.23)	25 (0–67)	0.17	−0.06 to 0.24
Publication bias					
Adjusting for publication bias^c^	26	0.09 (−0.01 to 0.19)	39 (3–62)	0.08	0.18–0.37
Sensitivity analyses					
Outlier excluded	24	0.06 (−0.01 to 0.13)	7 (0–40)	0.09	−0.01 to 0.13
Risk of bias^d^					
High risk	17	0.04 (−0.09 to 0.17)	47 (7–70)	0.52	NA
Some concerns	5	0.13 (0.08–0.18)	0 (0–79)	<0.001	NA
Low risk	3	0.19 (−0.37 to 0.76)	64 (0–90)	0.50	NA
Long-term					
6 to 9 months	17	0.14 (0.01–0.27)	39 (0–66)	0.03	−0.18 to 0.46
12 to 18 months^e^	10	0.38 (−0.21 to 0.98)	91 (85–94)	0.18	−1.54 to 2.30

CAU, care-as-usual; CI, confidence interval; NA, not applicable; PI, prediction interval; WL, waiting list.

a According to the random-effects model.

b *k* = 6 studies included individuals with depressive symptoms scoring above a cut-off on a standardized mental disorder symptom measure (of which *k* = 5 are on perinatal depression), and *k* = 1 study included adults with a clinical diagnosis.

c Egger's test was not significant (*p* = 0.74) and the number of imputed studies using Duvall and Tweedie trim-and-fill procedure was 26.

d The *p* value for the between-group effect sizes is not significant (*p* = 0.45).

e Of the *k* = 10 studies, only one study included 18 months follow-up data, the remaining studies reported 12 months follow-up data.

For the subgroup of patients with SMI (Boevink et al., 2016; Cook et al., 2012a; Corrigan et al., 2017; Davidson et al., 2004; Johnson et al., 2018; Kaplan et al., 2011; Mahlke et al., 2017; O'Connell et al., 2018; Pfeiffer et al., 2019; Rivera et al., 2007; Rogers et al., 2016; Russinova et al., 2014; Solomon & Draine, 1995), the effect size was significant, *g* *=* 0.18, 95% CI (0.10–0.26) (14 trials), but not for the six trials targeting individuals with depressive symptoms (Dennis, 2003; Dennis et al., 2009; Gjerdingen et al., 2013; Griffiths et al., 2012; Letourneau et al., 2011; Shorey et al., 2019), *g* *=* 0.02, 95% CI (−0.34 to 0.37). No significant effect sizes were observed in any of the examined specific outcomes: for *quality of life*, *g* *=* 0.08, 95% CI (−0.04 to 0.19), *social functioning*, *g* *=* 0.07, 95% CI (−0.05 to 0.18), and *loneliness*, *g* *=* 0.09, 95% CI (−0.05 to 0.23). Conducting subgroup analyses, we found no differences in the effect of PSIs among potential moderators (see Appendix I).

No indications of publication bias were observed, Egger's test, *p* = 0.74, see Figure J3 in Appendix J. When one outlier was removed (Salzer et al., 2016), the effect size remained significant, *g* *=* 0.06, 95% CI (−0.01 to 0.13). Subgroup analyses showed no differences in effects between RoB levels. Pooling the three trials rated at low risk resulted in a nonsignificant effect of *g* *=* 0.19, 95% CI (−0.37 to 0.76).

Long-term effects for all functional recovery outcomes demonstrated a significant effect size at six to nine months follow-up, *g* *=* 0.14, 95% CI (0.01-0.27) (17 trials). At 12 to 18 months follow-up, effects were nonsignificant, *g* *=* 0.38, 95% CI (−0.21 to 0.98).

## Discussion

In this comprehensive meta-analysis of 28 RCTs (*n* = 4152), PSIs for patients covering a broad spectrum of mental illnesses were associated with superior outcomes compared with control conditions regarding: (a) *clinical* recovery at post-test, and six to nine months follow-up; (b) *personal* recovery at post-test; and (c) *functional* recovery limited to six to nine months follow-up. When examining specific groups, we saw that specifically in the SMI patients – individuals with serious mental disorders – peer support was associated with significant superiority to control conditions at post-intervention across all three recovery categories. For the subgroup of individuals with elevated depressive symptoms – most of them being perinatal women – no significant effects were found in any of the recovery categories. Nonetheless, the number of trials targeting this group was small and nonsignificant results could be due to a lack of power. Also, the analyses for more category-specific outcomes within each main outcome category were exploratory due to the small number of studies. Only the effect size for *hope*, considered part of personal recovery, was significant.

We found no significant differences in the effect of PSIs among potential moderators (e.g. intervention delivery) for any of the outcomes, which could suggest that common values of peer support exceed disorder-specific needs and the intervention type. However, subgroup analyses should be considered with caution, since the number of trials for some categories was small and these analyses are likely underpowered. Accordingly, we could not analyze differences in effects between internet-based PSIs (2 trials) and traditional face-to-face interventions (16 trials; see Appendix I). Since the evidence-base for eHealth is increasing (Chan et al., 2022; Deady et al., 2017; Massoudi, Holvast, Bockting, Burger, & Blanker, 2019) and digital PSIs for individuals with SMI seem to be associated with positive changes for both clinical and psychosocial outcomes (Fortuna et al., 2020), the effectiveness for technology-based PSIs should be further investigated.

The pooled effect sizes, that were confirmed in sensitivity analyses, were small ranging from *g* *=* 0.15 for overall personal recovery to *g* *=* 0.19 for overall clinical recovery at post-test. A surprising finding was low to moderate heterogeneity, suggesting that the effects were consistent across wide-varying studies. However, due to the relatively large width of the 95% CIs, caution must be applied. Moreover, although the effect size for clinical recovery appeared to be more robust, the prediction intervals for personal and functional recovery suggested that the effects are considerably uncertain. In addition, the risk of bias was high for the majority of included studies and we could not reliably estimate its impact on the results of the meta-analysis.

Operating with a broad scope, including the largest number of trials on peer support to date, we found a significant though small effect size for *clinical* recovery. This was not detected in previous meta-analyses (Burke et al., 2019; Chien et al., 2019; Fuhr et al., 2014; Huang et al., 2020; Lloyd-Evans et al., 2014; Lyons et al., 2021; White et al., 2020), possibly due to lack power. Considering the efficacy of peer support for *personal* recovery, we confirmed and extended the results of previous meta-analyses (Bryan & Arkowitz, 2015; Burke et al., 2019; Fuhr et al., 2014; Lloyd-Evans et al., 2014; Lyons et al., 2021; White et al., 2020). So far, outcomes for *functional* recovery are scarcely addressed in peer support meta-analyses (Fuhr et al., 2014; Lyons et al., 2021). Whilst only valid for the subgroup SMI and long-term analysis, we found significant effect sizes for functional recovery, with *quality of life* as the most important outcome parameter. Overall, results indicate that peer support is of clinical relevance for individuals with mental illness, and not limited to reinforcing personal recovery following the generally accepted recovery-oriented approach (Leamy, Bird, Le Boutillier, Williams, & Slade, 2011; van Weeghel et al., 2019).

### Limitations

The results of this study should be considered with caution because of several important limitations. First, measures for clinical, personal, and functional recovery differed considerably across studies. Second, long-term effects were limited to smaller samples of trials up to 12 months follow-up. Third, a major limitation of this study is the high risk of bias for the majority of trials, with limited reporting for many of the risk of bias items. Since peer support has an informal nature, it is difficult to quantitatively analyze these interventions. An established protocol would help to quantify variables that could be evaluated in trials, but this would restrict the open nature of PSIs. Still, since peer support has been increasingly considered an essential element for recovery there have been attempts to structure and professionalize PSIs (Chinman et al., 2016; SAMHSA, 2015). However, doubts remain because the core of peer support is its naturalistic approach (Fortuna, Solomon, & Rivera, 2022). The feasibility, acceptability, and benefits of structuring and professionalizing PSIs need further investigation. To improve the quality of studies, future research should implement clinician-rated instruments and prospective registration in clinical trial registries. Finally, though comparing the efficacy of PSIs with clinical psychotherapies seems relevant for implementing or referring to PSIs in mental health care, the number of trials was too small to conduct a meta-analysis for RCTs with a clinician-led comparator.

## Conclusions

Engaging in a peer support intervention may be effective for reducing clinical mental illness symptoms, improving overall personal recovery, and more specifically hope. In particular for individuals with SMI, peer support demonstrated probable efficacy across the three recovery categories. Although the effects were small, peer support is a potentially cost-effective and relatively easy-to-implement intervention, and may complement professional treatment. Therapists, general practitioners, and employees of recovery-oriented services may refer their clients to peer support initiatives to expand the individuals' context to work on recovery when coping with mental illness.

## Supplementary material

For supplementary material accompanying this paper visit https://doi.org/10.1017/S0033291722002422. click here to view supplementary material
