# Supplementary material for: The effectiveness of peer support for individuals with mental illness: systematic review and meta-analysis
Source: Psychol Med. 2022 Sep 6;53(11):5332–41. doi: 10.1017/S0033291722002422 (PMC10476060; doi:10.1017/S0033291722002422)
Supplement: Supplementary file 1 [file S0033291722002422sup001.docx]

##

## **Appendices for the research article:**

Smit, D., Miguel, C., Vrijsen, J. N., Groeneweg, B., Spijker, J., & Cuijpers, P.

The effectiveness of peer support for individuals with mental illness:

Systematic review and meta-analysis.

## **Overview Appendices**

| **In-Text Citation** | **Content** |  |  | **Page number** |
| --- | --- | --- | --- | --- |
| Appendix A | Complete search strings per database (PubMed, Embase, and PsycINFO) | | | 2 |
| Appendix B | Definitions for the three main outcome categories:  clinical, personal, and functional recovery | | | 5 |
| Appendix C | Details for data extraction and calculating Risk of Bias scores | | | 8 |
| Appendix D | Selected Characteristics of Included Studies | | | 9 |
| Appendix E | References, list of included studies in the systematic review and meta-analyses | | | 14 |
| Appendix F | Narrative description of 3 RCTs with a clinician-led comparator | | | 17 |
| Appendix G | Risk of Bias Graphs | | | 18 |
| Appendix H | Risk of Bias rating per study: subdomains and overall rating | | | 19 |
| Appendix I | Subgroup analyses on moderators per outcome category:  Clinical, Personal, and Functional Recovery Outcomes, Hedges *g* | | | 21 |
| Appendix J | Figure J1, J2, and J3: Funnel Plots | | | 22 |
| Appendix K | Figure K3. Effect Sizes of Personal Recovery Outcomes | | | 24 |
|  | Figure K4. Effect Sizes of Functional Recovery Outcomes | | | 25 |

## **Appendix A**

**Complete search strings per database (PubMed, Embase, and PsycINFO)**

Search string used for PubMed, with keywords:

Block 1: Peer support;

Block 2: Mental health illness;

Filter: RCTs.

Block 1:

((Peer*[tiab] OR buddy[tiab] OR buddies[tiab] OR “mutual help”[tiab] OR “mutual support”[tiab] OR “social support”[tiab] OR “Peer-based*”[tiab] OR “Peer support*”[tiab] OR “Peer-led*”[tiab] OR “Peer-provided*”[tiab] OR “Peer-run*”[tiab] OR “Peer to peer*”[tiab]) AND (intervent*[tiab] OR therap*[tiab] OR coach*[tiab] OR counsel*[tiab] OR program*[tiab] OR service*[tiab] OR “Self-help group*”[tiab])) OR “Mutual Support*”[tiab] OR “Mutual help*”[tiab] OR “Shared medical appointment*”[tiab] OR “Patient to patient*”[tiab] OR “Peer self-management*”[tiab] OR “Support group*”[tiab] OR “Support program*”[tiab] OR “Support intervention*”[tiab] OR ((online[tiab] OR internet*[tiab]) AND (forum[tiab] OR group[tiab] OR communit*[tiab] OR discussion[tiab] OR board[tiab]) AND (support[tiab] OR “mutual help”[tiab] OR “peer”[tiab] OR “buddy”[tiab] OR “buddies”[tiab])) OR ((“Self-help groups”[MeSH] OR “Self-management”[MeSH]) AND (“Peer”[tiab] OR buddy[tiab] OR buddies[tiab] OR “mutual help”[tiab] OR “mutual support”[tiab]))

AND

Block 2:

“mental health*”[tiab] OR “Mental Health”[Mesh] OR “mental disorder*”[tiab] OR “Mental disorders”[Mesh] OR “mental diagnos*”[tiab] OR “mental symptom*”[tiab] OR “mentally ill*”[tiab] OR “mental illness*”[tiab] OR “mental problem*”[tiab] OR “mental disease*”[tiab] OR “psychological disorder*”[tiab] OR “psychological symptom*”[tiab] OR “psychological diagnos*”[tiab] OR “psychological illness*”[tiab] OR “psychological disease*”[tiab] OR “psychological problem*”[tiab] OR “psychological patient*”[tiab] OR “Psychiatric symptom*”[tiab] OR “psychiatric disorder*”[tiab] OR “psychiatric diagnos*”[tiab] OR “psychiatric illness*”[tiab] OR “psychiatric disease*”[tiab] OR “psychiatric problem*”[tiab] OR “psychiatric patients”[tiab] OR “behavioural disorder*”[tiab] OR “behavioral disorder*”[tiab] OR “behaviour disorder*”[tiab] OR “behavior disorder*”[tiab] OR “psychological distress*”[tiab] OR “Mental distress*”[tiab] OR “Mentally distress*”[tiab] OR “Mentally Ill Persons”[Mesh] OR “Axis I disorder*”[tiab] OR “Axis 1 disorder*”[tiab] OR “affective disorder*”[tiab] OR “anxiet*”[tiab] OR “agoraphob*”[tiab] OR “neurotic disorder*”[tiab] OR “obsessive compulsive*”[tiab] OR “ocd”[tiab] OR “panic*”[tiab] OR “phobi*”[tiab] OR “gad”[tiab] OR "bipolar*"[tiab] OR “eating disorder*”[tiab] OR “anorexi*”[tiab] OR “Bulimi*”[tiab] OR “Mood disorder*”[tiab] OR “depress*”[tiab] OR “dysthym*”[tiab] OR “personality disorder*”[tiab] OR "psychotic*"[tiab] OR “psychosis*”[tiab] OR “schizophren*”[tiab] OR “psychosis”[tiab] OR "somatoform disorder*"[tiab] OR “trauma*”[tiab] OR “posttraumatic stress”[tiab] OR “post-traumatic stress” OR "ptsd"[tiab]

With filter for RCTs

Search string used for PsycINFO, with keywords ^a^

Block 1: Peer support;

Block 2: Mental health illness;

Filter: RCTs.

*^a^ The double quotations (“) need to be entered in the search box at the PsycINFO website. These characters cannot be copy-pasted from a text document into the online search box because quotations are changed to italic characters.*

Block 1:

((Peer*.ti,ab. OR buddy.ti,ab. OR buddies.ti,ab. OR “mutual help”.ti,ab. OR “mutual support”.ti,ab. OR “social support”.ti,ab. OR “peer-based*”.ti,ab. OR “peer support*”.ti,ab. OR “Peer-led*”.ti,ab. OR “Peer-provided*”.ti,ab. OR “Peer-run*”.ti,ab. OR “Peer to peer*”.ti,ab.) AND (intervent*.ti,ab. OR therap*.ti,ab. OR coach*.ti,ab. OR counsel*.ti,ab. OR program*.ti,ab. OR service*.ti,ab. OR “Self-help group*”.ti,ab.)) OR

“Mutual Support*”.ti,ab. OR “Mutual help*”.ti,ab. OR “Shared medical appointment*”.ti,ab. OR “Patient to patient*”.ti,ab. OR “Peer self-management*”.ti,ab. OR “Support group*”.ti,ab. OR “Support program*”.ti,ab. OR “Support intervention*”.ti,ab.

OR ((online.ti,ab. OR internet*.ti,ab.) AND (forum.ti,ab. OR group.ti,ab. OR communit*.ti,ab. OR discussion.ti,ab. OR board.ti,ab.) AND (support.ti,ab. OR “mutual help”.ti,ab. OR “peer”.ti,ab. OR “buddy”.ti,ab. OR “buddies”.ti,ab.))

OR ((exp Social Support/ or exp Support Groups/ or exp Self-Help Techniques/ or exp Social Support/ or exp Support Groups/ or exp Online Community/ or exp Online Social Networks/ or exp Group Discussion/ or exp Social Groups/ or exp Social Group Work/ or exp Self-Management/) AND (exp Peers/ or Peer*.ti,ab. OR buddy.ti,ab. OR buddies.ti,ab. OR “mutual help”.ti,ab. OR “mutual support”.ti,ab.))

AND

Block 2:

exp Mental Health/ or exp Mental Disorders/ or exp Chronic Mental Illness or exp Psychiatric Patients/ or exp Psychiatric Symptoms/ or exp Personality Disorders/ or exp Psychodiagnosis/ or exp Mood Disorder/ or exp Psychopathology OR mental health*.ti,ab. OR mental disorder*.ti,ab. OR mental diagnos*.ti,ab. OR mental symptom*.ti,ab. OR mentally ill*.ti,ab. OR mental illness*.ti,ab. OR mental problem*.ti,ab. OR mental disease*.ti,ab. OR psychological disorder*.ti,ab. OR psychological symptom*.ti,ab. OR psychological diagnos*.ti,ab. OR psychological illness*.ti,ab. OR psychological disease*.ti,ab. OR psychological problem*.ti,ab. OR psychological patient*.ti,ab. OR Psychiatric symptom*.ti,ab. OR psychiatric disorder*.ti,ab. OR psychiatric diagnos*.ti,ab. OR psychiatric illness*.ti,ab. OR psychiatric disease*.ti,ab. OR psychiatric problem*.ti,ab. OR psychiatric patients.ti,ab. OR psychological distress*.ti,ab. OR Mental distress*.ti,ab. OR Mentally distress*.ti,ab. OR Axis I disorder*.ti,ab. OR Axis 1 disorder*.ti,ab. OR affective disorder*.ti,ab. OR anxiet*.ti,ab. OR agoraphob*.ti,ab. OR neurotic disorder*.ti,ab. OR obsessive compulsive*.ti,ab. OR ocd.ti,ab. OR panic*.ti,ab. OR phobi*.ti,ab. OR gad.ti,ab. OR bipolar*.ti,ab. OR eating disorder*.ti,ab. OR anorexi*.ti,ab. OR Bulimi*.ti,ab. OR Mood disorder*.ti,ab. OR depress*.ti,ab. OR dysthym*.ti,ab. OR personality disorder*.ti,ab. OR psychotic*.ti,ab. OR psychosis*.ti,ab. OR schizophren*.ti,ab. OR psychosis.ti,ab. OR somatoform disorder*.ti,ab. OR trauma*.ti,ab. OR posttraumatic stress.ti,ab. OR post-traumatic stress.ti,ab OR ptsd.ti,ab.

With filter for clinical trials

Search string used for Embase, with keywords:

Block 1: Peer support;

Block 2: Mental health illness;

Filter: RCTs.

Block 1:

((Peer*:ti,ab OR buddy:ti,ab OR buddies:ti,ab OR ‘mutual help’:ti,ab OR ‘mutual support’:ti,ab OR ‘social support’:ti,ab OR ‘Peer-based*’:ti,ab OR ‘Peer support*’:ti,ab OR ‘Peer-led*’:ti,ab OR ‘Peer-provided*’:ti,ab OR ‘Peer-run*’:ti,ab OR ‘Peer to peer*’:ti,ab) AND (intervent*:ti,ab OR therap*:ti,ab OR coach*:ti,ab OR counsel*:ti,ab OR program*:ti,ab OR service*:ti,ab OR ‘Self-help group*’:ti,ab)) OR ‘Mutual Support*’:ti,ab OR ‘Mutual help*’:ti,ab OR ‘Shared medical appointment*’:ti,ab OR ‘Patient to patient*’:ti,ab OR ‘Peer self-management*’:ti,ab OR ‘Support group*’:ti,ab OR ‘Support program*’:ti,ab OR ‘Support intervention*’:ti,ab OR ((online:ti,ab OR internet*:ti,ab) AND (forum:ti,ab OR group:ti,ab OR communit*:ti,ab OR discussion:ti,ab OR board:ti,ab) AND (support:ti,ab OR ‘mutual help’:ti,ab OR ‘peer’:ti,ab OR ‘buddy’:ti,ab OR ‘buddies’:ti,ab))

OR ((‘Self care’/exp OR ‘psychosocial care’/exp OR ‘self help’/exp) AND (‘Peer’:ti,ab OR buddy:ti,ab OR buddies:ti,ab OR ‘mutual help’:ti,ab OR “mutual support”:ti,ab))

AND

Block 2:

‘mental health*’:ti,ab OR ‘mental health’/exp OR ‘mental disorder*’:ti,ab OR ‘mental disease’/exp OR ‘mental diagnos*’:ti,ab OR ‘mental symptom*’:ti,ab OR ‘mentally ill*’:ti,ab OR ‘mental illness*’:ti,ab OR ‘mental problem*’:ti,ab OR ‘mental disease*’:ti,ab OR ‘psychological disorder*’:ti,ab OR ‘psychological symptom*’:ti,ab OR ‘psychological diagnos*’:ti,ab OR ‘psychological illness*’:ti,ab OR ‘psychological disease*’:ti,ab OR ‘psychological problem*’:ti,ab OR ‘psychological patient*’:ti,ab OR ‘Psychiatric symptom*’:ti,ab OR ‘psychiatric disorder*’:ti,ab OR ‘psychiatric diagnos*’:ti,ab OR ‘psychiatric illness*’:ti,ab OR ‘psychiatric disease*’:ti,ab OR ‘psychiatric problem*’:ti,ab OR ‘psychiatric patients’:ti,ab OR ‘behavioural disorder*’:ti,ab OR ‘behavioral disorder*’:ti,ab OR ‘behaviour disorder*’:ti,ab OR ‘behavior disorder*’:ti,ab OR ‘psychological distress*’:ti,ab OR ‘Mental distress*’:ti,ab OR ‘Mentally distress*’:ti,ab OR ‘mental patient’/exp OR ‘Axis I disorder*’:ti,ab OR ‘Axis 1 disorder*’:ti,ab OR ‘affective disorder*’:ti,ab OR ‘anxiet*’:ti,ab OR ‘agoraphob*’:ti,ab OR ‘neurotic disorder*’:ti,ab OR ‘obsessive compulsive*’:ti,ab OR ‘ocd’:ti,ab OR ‘panic*’:ti,ab OR ‘phobi*’:ti,ab OR ‘gad’:ti,ab OR ‘bipolar*’:ti,ab OR ‘eating disorder*’:ti,ab OR ‘anorexi*’:ti,ab OR ‘Bulimi*’:ti,ab OR ‘Mood disorder*’:ti,ab OR ‘depress*’:ti,ab OR ‘dysthym*’:ti,ab OR ‘personality disorder*’:ti,ab OR ‘psychotic*’:ti,ab OR ‘psychosis*’:ti,ab OR ‘schizophren*’:ti,ab OR ‘psychosis’:ti,ab OR ‘somatoform disorder*’:ti,ab OR ‘trauma*’:ti,ab OR ‘posttraumatic stress’:ti,ab OR ‘post-traumatic stress’ OR ‘ptsd’:ti,ab

With filter for RCTs

## **Appendix B**

**Definitions for the three main outcome categories: clinical, personal, and functional recovery**

Recovery is a complex and multidimensional concept, and has been defined in various ways (Bellack, 2006; Jääskeläinen et al., 2013; Whitley & Drake, 2010). Three types of recovery can be differentiated, that are complementary aspects of recovery rather than mutual exclusive categories. Recovery can be seen as both outcome and process (Roosenschoon, Kamperman, Deen, Weeghel, & Mulder, 2019). For evaluating the effects of peer support interventions, we will examine the following three main types of outcomes:

1. Clinical or symptomatic recovery: the degree of psychiatric symptomatology (Slade et al., 2014; Van Eck, Burger, Vellinga, Schirmbeck, & de Haan, 2018). This does not equate with symptomatic remission (the absence of a sustained reduction in symptoms).
2. Personal recovery, or sometimes referred to as subjective recovery (Mueser et al., 2006), highlights the personal nature of the recovery process; a term that originated among people with lived experience of mental illness (Deegan, 2002; Leamy, Bird, Le Boutillier, Williams, & Slade, 2011; Mead & Copeland, 2000). It includes components such as spirituality, empowerment, actively accepting the illness, and also finding hope, re-establishing a positive identity, developing meaning in life, overcoming stigma, taking control of one’s own life, and having supporting relationships (Cavelti, Kvrgic, Beck, Kossowsky, & Vauth, 2012). In a shorter definition, it concerns the extents of perceived recovery, sense of purpose, and personal agency (Mueser et al., 2006). To summarize the key elements of personal recovery, various authors use the acronym CHIME: Connectedness; Hope and Optimism about the future; Identity; Meaning in life; and Empowerment (Leamy et al., 2011). According to a recent systematic review and meta-analyses, “Difficulties and trauma” should be added, and the person’s choice, risk taking, and coping with challenges should be emphasized in this framework (van Weeghel, van Zelst, Boertien, & Hasson-Ohayon, 2019).
3. Functional recovery or objective recovery (Mueser et al., 2006): the degree of vocational and social functioning, such as acting according to age-appropriate role expectations, the performance of daily living tasks without supervision, engagement in social interactions (Robinson, Woerner, McMeniman, Mendelowitz, & Bilder, 2004), and the degree of independence with regard to housing (Harvey & Bellack, 2009; Whitley & Drake, 2010). Functional recovery thus concerns functional outcomes rather than functional capacity (Carrión et al., 2013; Patterson & Mausbach, 2010). Some studies interpret functional recovery with functional remission (Harvey & Bellack, 2009), others consider it part of clinical recovery (Liberman & Kopelowicz, 2002; Slade et al., 2012)

**References used for defining recovery outcome categories**

Bellack, A. S. (2006). Scientific and consumer models of recovery in schizophrenia: concordance, contrasts, and implications.

Carrión, R. E., McLaughlin, D., Goldberg, T. E., Auther, A. M., Olsen, R. H., Olvet, D. M., . . . Cornblatt, B. A. (2013). Prediction of functional outcome in individuals at clinical high risk for psychosis. *JAMA psychiatry, 70*(11), 1133-1142.

Cavelti, M., Kvrgic, S., Beck, E. M., Kossowsky, J., & Vauth, R. (2012). Assessing recovery from schizophrenia as an individual process. A review of self-report instruments. *European psychiatry, 27*(1), 19-32.

Deegan, P. E. (2002). Recovery as a self-directed process of healing and transformation. *Occupational Therapy in Mental Health, 17*(3-4), 5-21.

Harvey, P. D., & Bellack, A. S. (2009). Toward a terminology for functional recovery in schizophrenia: is functional remission a viable concept? *Schizophrenia bulletin, 35*(2), 300-306.

Jääskeläinen, E., Juola, P., Hirvonen, N., McGrath, J. J., Saha, S., Isohanni, M., . . . Miettunen, J. (2013). A systematic review and meta-analysis of recovery in schizophrenia. *Schizophrenia bulletin, 39*(6), 1296-1306.

Leamy, M., Bird, V., Le Boutillier, C., Williams, J., & Slade, M. (2011). Conceptual framework for personal recovery in mental health: systematic review and narrative synthesis. *The British Journal of Psychiatry, 199*(6), 445-452.

Liberman, R. P., & Kopelowicz, A. (2002). Recovery from schizophrenia: a challenge for the 21st century. *International Review of Psychiatry, 14*(4), 245-255.

Mead, S., & Copeland, M. E. (2000). What recovery means to us: Consumers' perspectives. *Community mental health journal, 36*(3), 315-328.

Mueser, K. T., Meyer, P. S., Penn, D. L., Clancy, R., Clancy, D. M., & Salyers, M. P. (2006). The Illness Management and Recovery program: rationale, development, and preliminary findings. *Schizophrenia bulletin, 32*(suppl_1), S32-S43.

Patterson, T. L., & Mausbach, B. T. (2010). Measurement of functional capacity: a new approach to understanding functional differences and real-world behavioral adaptation in those with mental illness. *Annual review of clinical psychology, 6*, 139-154.

Robinson, D. G., Woerner, M. G., McMeniman, M., Mendelowitz, A., & Bilder, R. M. (2004). Symptomatic and functional recovery from a first episode of schizophrenia or schizoaffective disorder. *American Journal of Psychiatry, 161*(3), 473-479.

Roosenschoon, B.-J., Kamperman, A. M., Deen, M. L., Weeghel, J. v., & Mulder, C. L. (2019). Determinants of clinical, functional and personal recovery for people with schizophrenia and other severe mental illnesses: A cross-sectional analysis. *PloS one, 14*(9), e0222378.

Slade, M., Amering, M., Farkas, M., Hamilton, B., O'Hagan, M., Panther, G., . . . Whitley, R. (2014). Uses and abuses of recovery: implementing recovery‐oriented practices in mental health systems. *World Psychiatry, 13*(1), 12-20.

Slade, M., Leamy, M., Bacon, F., Janosik, M., Le Boutillier, C., Williams, J., & Bird, V. (2012). International differences in understanding recovery: systematic review. *Epidemiology and psychiatric sciences, 21*(4), 353-364.

Van Eck, R. M., Burger, T. J., Vellinga, A., Schirmbeck, F., & de Haan, L. (2018). The relationship between clinical and personal recovery in patients with schizophrenia spectrum disorders: a systematic review and meta-analysis. *Schizophrenia Bulletin, 44*(3), 631-642.

van Weeghel, J., van Zelst, C., Boertien, D., & Hasson-Ohayon, I. (2019). Conceptualizations, assessments, and implications of personal recovery in mental illness: A scoping review of systematic reviews and meta-analyses. *Psychiatric Rehabilitation Journal, 42*(2), 169.

Whitley, R., & Drake, R. E. (2010). Recovery: a dimensional approach. *Psychiatric services, 61*(12), 1248-1250.

## **Appendix C**

**Details for data extraction and calculating Risk of Bias scores**

Decision tool for data extraction of control conditions:

CAU control groups were prioritized for extraction over WL control groups when multiple control groups were available.

Decision tool for data extraction of multiple available instruments within one outcome category:

For (1) clinical recovery we chose (1a) a specific-disorder instrument over a transdiagnostic instrument if all participants were recruited based on one specific disorder. We chose a transdiagnostic instrument if multiple disorders were included. Furthermore, we chose (1b) a clinician-rated instrument over a self-reported measure. For (2) personal and functional recovery we chose instruments regarding global functioning in these domains over a more specific instrument. For example, we used data for personal and functional recovery for a more general instrument, the Recovery Assessment Schedule (RAS) over instruments for empowerment specifically (e.g., Empowerment Scale), and we extracted data for Quality of Life (e.g., Lehman's Quality of Life) rather than data of measurements on -often considered- a subcategory of Quality of Life, social support.

Details for calculating Risk of Bias score for domain 4

Trials that used both self-report measures and (blinded) clinician rated instruments were rated at low risk and some concerns for bias for domain 4 (inappropriate measurement of the outcome) if the type of measurement instrument differed per outcome category. For example, when authors used a self-report measure for clinical recovery and a blinded-clinician rated instrument for personal recovery, domain 4 was rated at some concerns for bias for the clinical recovery outcome category and at low risk for bias for the outcome category personal recovery.

Details for calculating an overall Risk of Bias score:

Overall high risk of bias was determined when any of the domains had a high risk score, or if 4 domains were rated as having ‘some concerns’. An overall low risk score was given when 4 domains were rated as low risk. An overall rating of some concerns was given in the remaining situations.

Details for data extraction per outcome category:

For clinical recovery instruments, we extracted means (*SD*) values of baseline, the primary end point and longest follow-up measurement for both the intervention and the control groups including sample sizes. For personal and functional recovery instruments, we extracted posttest and follow-up data when available for both intervention and the control groups.

## **Appendix D**

**Selected Characteristics of Included Studies**

| Author  and  publication year | Country | Population and Diagnoses | Subgroup | % Female | Age  (*M*) | Recruit-  ment ^a^ | Clinical diagnosis or cut-off | Sample size at post for clinical outcome: intervention/control | Intervention (name or reference in paper; structure; delivery; format; duration) | Control  condition | Outcomes | Assessments | Post  (months) | Long-term Follow-up (months) | Overall  Risk of Bias rating |
| --- | --- | --- | --- | --- | --- | --- | --- | --- | --- | --- | --- | --- | --- | --- | --- |
| Boevink 2016 | NL | SMI: 40.5% Non-affective psychotic disorder; 15.9% Affective disorder; 15% Personality disorder; 25% Other. | NA | 47.4 | 43.9 | Clinical (Inpatients + Outpatients) | Diagnosis | 65/73 | Peer-led structured face-to-face group intervention; 12 months (2-hour sessions, biweekly) | WL | 1) Clinical recovery (overall [transdiagnostic] clinical symptoms)  2) Personal recovery (empowerment)  3) Functional recovery (Quality of Life) | 1) Community Assessment of Psychich Experiences (CAPE)  2) Boston Empowerment Scale  3) Lancashire Quality of Life Profile (LQOLP) | 12 | NA | High Risk |
| Castelein 2008 | NL | SMI: Psychosis (Schizophrenia 74.5%; Other 25.5%). | NA | 34.5 | 38.55 | Clinical (Not specified) | Diagnosis | 56/50 | Peer-led unstructured face-to-face group intervention; 8 months (1.5-hour sessions, biweekly) | WL | 1) Personal recovery (empowerment)  2) Functional recovery (Quality of Life) | 1) Mental Health Confidence Scale (MHSC)  2) The World Health Organisation Quality of Life (WHOQOL) | 8 | NA | Some concerns |
| Cook 2012a | USA | SMI: Bipolar disorder 38%; Depressive disorder 25%; other 13%; Schizophrenia 12%; Schizoaffective disorder 10%. | NA | 66 | 45.8 | Mixed | Diagnosis | 224/234 | Wellness Recovery Action Plan (WRAP): Peer-led structured face-to-face group intervention; 2 months (2.5-hour sessions, weekly) | WL | 1) Clinical recovery (overall [transdiagnostic] clinical symptoms)  2) Personal recovery (overall personal recovery)  3) Functional recovery (Quality of Life) | 1) Brief Symptom Inventory (BSI)  2) Recovery Assessment Scale (RAS)  3) The World Health Organisation Quality of Life (WHOQOL) | 2 | 8 | High Risk |
| Cook 2012b | USA | SMI: Bipolar disorder 39.5%; Depressive disorder 18%; Schizophrenia 15.4%, Schizo-affective disorder 5.4%; Other 8.6%. | NA | 55.6 | 42.8 | Mixed | Diagnosis | 170/172 | Building Recovery  of Individual Dreams and Goals through Education and Support (BRIDGES): Peer-led structured face-to-face group intervention; 2 months (2.5-hour sessions, weekly) | WL | 1) Personal recovery (overall personal recovery) | 1) Recovery Assessment Scale (RAS) | 3.5 | 9 | High Risk |
| Corrigan 2017 | USA | SMI: Major Depressive Disorder 49%; Bipolar disorder 17%; Anxiety disorder 12% Schizophrenia 10%. | Yes: Homeless African Americans | 39.0 | 52.88 | Mixed | Diagnosis | 34/33 | Peer Navigator Program (PNP): Peer-led unstructured face-to-face individual intervention; duration not specified (weekly with flexible frequency) | CAU | 1) Clinical recovery (overall [transdiagnostic] clinical symptoms)  2) Personal recovery (overall personal recovery)  3) Functional recovery (Quality of Life) | 1) Texas Christian University Health Form (TCU HF)  2) Recovery Assessment Scale (RAS)  3) Short Form 36 Health Survey (SF-36) | 8 | 12 | Some concerns |
| Corrigan 2018 | USA | SMI: Major Depressive Disorder 68%; Anxiety 21%; Bipolar disorder 7%; Other. | Yes: Latinos | 58.5 | 45.65 | Clinical (Not specified) | Diagnosis | 55/55 | Peer Navigator Program (PNP): Peer-led unstructured face-to-face individual intervention; 6 months (weekly with flexible frequency) | CAU | 1) Personal recovery (overall personal recovery)  2) Functional recovery (Quality of Life) | 1) Recovery Assessment Scale (RAS)  2) Quality of Life Scale (QLS) | 8 | 12 | High Risk |
| Craig 2004 | UK | SMI: Paranoid schizophrenia 87%; drug/alcohol abuse 29%. | Yes: Long-term unemployed individuals | 33.3 | 37.6 | Clinical (Outpatients) | Diagnosis | 24/21 | Consumer-employee assistant health case management: Peer-led unstructured face-to-face individual intervention; duration not specified | CAU | 1) Functional recovery (functioning) | 1) Life Skill Profile (LSP) | 12 | NA | Some concerns |
| Author  and  publication year | Country | Population and Diagnoses | Subgroup | % Female | Age  (*M*) | Recruit-  ment ^a^ | Clinical diagnosis or cut-off | Sample size at post for clinical outcome: intervention/control | Intervention (name or reference in paper; structure; delivery; format; duration) | Control  condition | Outcomes | Assessments | Post  (months) | Long-term Follow-up (months) | Overall  Risk of Bias rating |
| Davidson 2004 | USA | SMI: Psychotic disorder 50%; Affective disorder 34%; Anxiety disorder 2%; Other Axis-I disorder 1%; Unknown 12%. | NA | 43 | 42 | Clinical (Outpatients) | Diagnosis | 95/70 | The Partnership Project: Peer-led unstructured face-to-face individual intervention; 9 months (2 to 4-hour session, weekly) | CAU | 1) Clinical recovery (overall [transdiagnostic] clinical symptoms)  2) Personal recovery (overall personal recovery)  3) Functional recovery (functioning) | 1) Brief Psychiatric Rating Scale (BPRS)  2) Wellbeing Scale (WBS)  3) Global Assessment of Functioning (GAF) | 9 | NA | High Risk |
| Dennis 2003 | Canada | Depression: Postpartum depression | Yes: Perinatal depression | 100 | NA (76.5% between 25-34 years) | Other | Cut-off | 20/22 | Mother-to-mother telephone based peer support: Peer-led unstructured telephone individual intervention; duration not specified | CAU | 1) Clinical recovery (depression)  2) Functional recovery (Loneliness) | 1) Edinburgh Postnatal Depression Scale (EPSD)  2) University of California Los Angeles Loneliness Scale (UCLA LS) | 2 | NA | Low Risk |
| Dennis 2009 | Canada | Depression: Postpartum depression | Yes: Perinatal depression | 100 | NA (78% between 20-34 years) | Other | Cut-off | 297/315 | Mother-to-mother telephone based peer support: Peer-led unstructured telephone individual intervention; 3 months (flexible frequency) | CAU | 1) Clinical recovery (depression)  2) Functional recovery (Loneliness) | 1) Edinburgh Postnatal Depression Scale (EPSD)  2) University of California Los Angeles Loneliness Scale (UCLA LS) | 3 | 6 | Some concerns |
| Field 2013  *Not included in MA* | USA | Depression: Prenatal depression | Yes: Perinatal depression | 100 | 24.9 | Other | Diagnosis | 22/22 | Peer support group: Peer-led unstructured face-to-face group intervention: 3 months (sessions weekly) | Clinician-led control group | 1) Clinical recovery (depression) | 1) Center for Epidemiologic Studies Depression (CES-D) | 3 | NA | High Risk |
| Gjerdingen 2013 | USA | Depression: Postpartum depression | Yes: Perinatal depression | 100 | 29.7 | Other | Cut-off | 11/14 | Peer telephone support: Peer-led unstructured telephone individual intervention; 3 months (flexible frequency) | CAU | 1) Clinical recovery (depression)  2) Functional recovery (Quality of Life) | 1) Center for Epidemiologic Studies Depression (CES-D)  2) EuroQoL 5D (EQ-5D) | 3 | 6 | High Risk |
| Griffiths 2012 | Australia | Depression: Diagnoses not specified | NA | 61 | 44.6 | General population | Cut-off | 52/71 | Wellbeing board, a moderated internet support group: Peer-led unstructured internet group intervention; 3 months (minimum of 2 logins weekly) | Attention control | 1) Clinical recovery (depression)  2) Personal recovery (empowerment)  3) Functional recovery (Quality of Life) | 1) Center for Epidemiologic Studies Depression (CES-D)  2) Empowerment Scale (subscale power-powerlessness)  3) EUROHIS QOL 8-item index (EUROHIS QOL) | 3 | 6 | High Risk |
| Johnson 2018 | UK | SMI (PS/CTR): Depression 23/25%; Schizophrenia or Schizoaffective disorder 13/15%; Bipolar 13/12%; Borderline 8/10%; Other Psychosis 6/4%. | NA | 60 | 40 | Inpatients | Diagnosis | 218/216 | Peer-supported self-management intervention, based on a recovery workbook: Peer-led structured face-to-face individual intervention; 4 months (1-hour sessions, weekly) | CAU | 1) Clinical recovery (overall [transdiagnostic] clinical symptoms)  2) Personal recovery (overall personal recovery)  3) Functional recovery (loneliness) | 1) Brief Psychiatric Rating Scale (BPRS)  2) Questionnaire about the Process of Recovery (QPR)  3) University of California Los Angeles Loneliness Scale (UCLA LS) | 4 | 18 | High Risk |
| Kaplan 2011 | USA | SMI: Schizophrenia Spectrum 22.41%; Affective disorder 77.59%. | NA | 65.67 | 47 | Mixed | Diagnosis | 99/100 | An unmoderated internet support group: Peer-led unstructured online group intervention; duration not specified (flexible frequency) | WL | 1) Clinical recovery (overall [transdiagnostic] clinical symptoms)  2) Personal recovery (overall personal recovery)  3) Functional recovery (Quality of Life) | 1) Hopkins Symptoms Checklist-58 (HSCL-58)  2) Recovery Assessment Scale (RAS)  3) Quality of Life Lehman (QoL Lehman) | 4 | 12 | Some concerns |
| Author  and  publication year | Country | Population and Diagnoses | Subgroup | % Female | Age  (*M*) | Recruit-  ment ^a^ | Clinical diagnosis or cut-off | Sample size at post for clinical outcome: intervention/control | Intervention (name or reference in paper; structure; delivery; format; duration) | Control  condition | Outcomes | Assessments | Post  (months) | Long-term Follow-up (months) | Overall  Risk of Bias rating |
| Letourneau 2011 | Canada | Depression: Postpartum depression | Yes: Perinatal | 100 | Majority 26-35 years. | Mixed | Cut-off | 23/28 | Home-based peer support intervention: Peer-led structured face-to-face and telephone individual intervention; 3 months (flexible frequency) | WL | 1) Clinical recovery (depression)  2) Functional recovery (functioning) | 1) Edinburgh Postnatal Depression Scale (EPSD)  2) Social Provision Scale (SPS) | 3 | NA | High Risk |
| Ludman 2007 | USA | Depression: Dysthymia 79%; Major depressive disorder 55%; Panic disorder 33%; Generalised anxiety disorder 28%; Borderline personality disorder 13%. | NA | 72 | 50.2 | Clinical (Inpatients+ Outpatients) | Diagnosis | 20/21 | Chronic disease self-management program: Peer-led structured face-to-face group intervention; 1.5 month (sessions weekly) | Other inactive control + Clinician-led control group | 1) Clinical recovery (overall [transdiagnostic] clinical symptoms) | 1) Structured Clinical Interview for  DSM-IV (SCID) | 12 | NA | High Risk |
| Mahlke 2017 | Germany | SMI: Unipolar depression 25%; Personality disorder 23%; Schizophrenia 22%; Bipolar disorder 15%; Schizoaffective disorder 6%; Other/NA both 5%. | NA | 57 | 41.48 | Clinical (Inpatients+ Outpatients) | Diagnosis | 61/42 | Peer-led unstructured face-to-face individual intervention; 6 months (flexible, in principle 1-hour sessions, biweekly) | CAU | 1) Clinical recovery (overall [transdiagnostic] clinical symptoms)  2) Personal recovery (Empowerment)  3) Functional recovery (Quality of Life) | 1) Clinical Global Impression Scale (CGI)  2) General Self-efficacy Scale (GSE)  3) EuroQoL 5D (EQ-5D) | 6 | 12 | High Risk |
| Matthews 2018  *Not included in MA* | USA | Hoarding disorder | Yes: Depression (Anxiety) | 74.5 | 58.95 | Mixed | Cut-off | 163/160 | Group Peer Facilitated Therapy (G-PFT): Peer-led unstructured face-to-face group + telephone individual intervention; 5 months (sessions approximately weekly) | Clinician-led Cognitive Behavioural Therapy | 1) Clinical recovery (overall [transdiagnostic] clinical symptoms) 2) Functional recovery (functioning) | 1) Saving Inventory-Revised (SI-R) 2) Activities of Daily Living Scale  in Hoarding Disorder (ADL-H). | 5 | 8 | High Risk |
| O’Connell 2018 | USA | SMI (PSI/CTR): Psychotic disorder 72/78%; Mood disorder 28/22%. | NA | 50 | 40.1 | Clinical (Inpatients) | Diagnosis | 34/29 | Recovery mentor: Peer-led unstructured face-to-face and/or telephone individual intervention; up to 9 months (flexible frequency, recommended weekly sessions) | CAU | 1) Clinical recovery (overall [transdiagnostic] clinical symptoms)  2) Functional recovery (Quality of Life) | 1) Brief Psychiatric Rating Scale (BPRS)  2) Short Form 36 Health Survey, 2 items on social functioning (SF-36) | 9 | NA | High Risk |
| Pfeiffer 2019 | USA | SMI: Unipolar mood disorder 58%; Bipolar mood disorder 12%; Schizophrenia 4%; Anxiety disorder 4%; Substance use disorder 6%; Personality disorder 10%; Other 5%. | NA | 53 | 34 | Clinical (Inpatients) | Other: Medical record documentation or suicidal ideation om Beck Scale for Suicidal Ideation ≥ 5. | 24/31 | Peers for Valued Living (PREVAIL): Peer-led structured mixed (primarily face-to-face with supporting text messages, mail, telephone) individual intervention; 3 months (flexible frequency, encouraged (bi)weekly) | CAU | 1) Clinical recovery (overall [transdiagnostic] clinical symptoms)  2) Personal recovery (Hope)  3) Functional recovery (functioning) | 1) Beck Scale for Suicide Ideation (BSSI)  2) Hope Scale (HS)  3) NIH Toolbox Adult Social Relationship Scales (NIH Toolbox) | 3 | 6 | High Risk |
| Author  and  publication year | Country | Population and Diagnoses | Subgroup | % Female | Age  (*M*) | Recruit-  ment ^a^ | Clinical diagnosis or cut-off | Sample size at post for clinical outcome: intervention/control | Intervention (name or reference in paper; structure; delivery; format; duration) | Control  condition | Outcomes | Assessments | Post  (months) | Long-term Follow-up (months) | Overall  Risk of Bias rating |
| Ranzenhofer 2020 | USA | Other: Eating disorders: Anorexia Nervosa 65%; Atypical Anorexia Nervosa 10%; Boulimia Nervosa 20%; Binge Eating Disorder 5%. | NA | 100 | 27.48 | Clinical (Inpatients + Outpatients) | Diagnosis | 18/20 | Peer mentorship: Peer-led mixed (face-to-face or online) individual intervention (structure not specified); 6 months (1 hour sessions, weekly) | WL | 1) Clinical recovery (overall [transdiagnostic] clinical symptoms)  2) Functional recovery (Quality of Life) | 1) Eating Pathology Symptoms Inventory (EPSI)  2) Eating Disorder Quality of Life (ED QOL) | 6 | 6 | Low risk |
| Rivera 2007 | USA | SMI: Schizophrenia 29%; Schizoaffective disorder 20%; Bipolar disorder 26%; Depressive disorder 22%. | NA | 49 | 38.3 | Clinical (Inpatients) | Diagnosis | 65/65 | Consumer assisted case management: Peer-led unstructured blended individual and group face-to-face intervention; 6 months (flexible frequency) | CAU | 1) Clinical recovery (overall [transdiagnostic] clinical symptoms)  2) Functional recovery (Quality of Life) | 1) Brief Symptom Inventory (BSI)  2) Quality of Life Lehman (QoL Lehman) | 6 | 12 | High risk |
| Rogers 2016 | USA | SMI: Diagnoses not specified | Yes: Civil Committed | 55.75 | 39.67 | Clinical (Inpatients) | Other: Adjudicated by the state court | 25/50 (high level of engagement)  28/50 (low level of engagement) | Peer support specialists: Peer-led unstructured face-to-face individual intervention; 6 months (sessions weekly) | CAU | 1) Clinical recovery (overall [transdiagnostic] clinical symptoms)  2) Personal recovery (overall personal recovery)  3) Functional recovery (Quality of Life) | 1) Behavior And Symptom Identification Scale 24 (BASIS-24)  2) Recovery Assessment Scale (RAS)  3) Quality of Life Lehman (QoL Lehman) | 6 | 6 | High risk |
| Rüsch 2014 | Switzerland | SMI: Depressive disorder 56%; Schizophrenia spectrum disorder 32%; Bipolar disorder 32% (including overlap). | NA | 59 | 41.95 | Mixed | Diagnosis | 39/47 | Coming Out Proud: Peer-led structured face-to-face group intervention; 0.75 months (2-hours sessions, weekly) | CAU | 1) Personal recovery (Empowerment) | 1) Empowerment Scale (ES) | 0.75 | NA | High risk |
| Russinova 2014 | USA | SMI: Schizophrenia Spectrum Disorder 34%; Bipolar disorder 33%; Depressive disorder 26%; Other 7%. | NA | 68 | Most participant were older than 40 (N = 556, 68%) | Clinical (Outpatients) | Diagnosis | 40/42 | Antistigma photovoice program: Peer-led structured face-to-face group intervention; 3 months (1.5 hours-sessions, weekly) | WL | 1) Clinical recovery (depression)  2) Personal recovery (overall personal recovery) | 1) Center for Epidemiologic Studies Depression (CES-D)  2) Personal Growth and Recovery Scale (PGRS) | 5.3 | NA | Some concerns |
| Salzer 2016 | USA | SMI: Schizophrenia; Bipolar disorder; Major Depression (% NA). | NA | 46.5 | 48.7 | Clinical (Outpatients) | Diagnosis | 50/49 | Peer-delivered Core Centre Independent Living: Peer-led structured face-to-face and telephone individual intervention; 6 months (flexible frequency) | CAU | 1) Personal recovery (overall personal recovery)  2) Functional recovery (Quality of Life) | 1) Recovery Assessment Scale (RAS)  2) Quality of Life Lehman (QoL Lehman) | 6 | 12 | High risk |
| Shorey 2019 | Singapore | Depression: Postnatal depression | Yes: Perinatal depression | 100 | 32.1 | Clinical (Other) | Cutt-off | 56/58 | Technology-based peer-support intervention program: Peer-led unstructured mixed (email, telephone, text messages) individual intervention; 1 month (flexible frequency, on average weekly sessions) | CAU | 1) Clinical recovery (depression)  2) Functional recovery (functioning) | 1) Edinburgh Postnatal Depression Scale (EPSD)  2) Perceived Social Support for Parenting (PSSP) | 1 | NA | Low risk |
| Author  and  publication year | Country | Population and Diagnoses | Subgroup | % Female | Age  (*M*) | Recruit-  ment ^a^ | Clinical diagnosis or cut-off | Sample size at post for clinical outcome: intervention/control | Intervention (name or reference in paper; structure; delivery; format; duration) | Control  condition | Outcomes | Assessments | Post  (months) | Long-term Follow-up (months) | Overall  Risk of Bias rating |
| Solomon 1995 | USA | SMI: Schizophrenia 86%; Major affective disorder 13%. | NA | 48 | 37.09 | Clinical (Outpatients) | Diagnosis | 48/48 | Consumer case management: Peer-led unstructured face-to-face individual intervention; 12 months (flexible frequency) | Active control | 1) Clinical recovery (overall [transdiagnostic] clinical symptoms)  2) Functional recovery (Quality of Life) | 1) Brief Psychiatric Rating Scale (BPRS)  2) Quality of Life Lehman (QoL Lehman) | 12 | 24 | High risk |
| Van Gestel-Timmermans 2012 | NL | SMI: Psychosis 33.46%; Affective disorder 36.5%; Anxiety disorder 22.47%; Personality disorder 32.02%. | NA | 66.02 | 43.49 | Mixed | Diagnosis | 136/117 | Recovery is up to you: Peer-led structured face-to-face group intervention; 3 months (2-hours sessions, weekly) | WL | 1) Personal recovery (Empowerment)  2) Functional recovery (Quality of Life) | 1) Netherlands Empowerment List (NEL)  2) Manchester Short Assessment of Quality of Life | 3 | 6 | High risk |

*Note.* Abbreviations: CAU = care as usual; CTRL = Control; MA = Meta-Analysis; NA = Not Applicable; PSI = Peer Support Intervention; SMI = Serious Mental Illness; WL = Waiting List.
^a^ Studies with mixed recruitment included both a clinical group (inpatients and/or outpatients) and individuals recruited in the general population.

**Appendix E**

**References, list of included studies in the systematic review and meta-analyses**

List of studies included in the meta-analysis (n = 28):

1. Boevink, W., Kroon, H., van Vugt, M., Delespaul, P., & van Os, J. (2016). A user-developed, user run recovery programme for people with severe mental illness: A randomised control trial. Psychosis, 8, 287-300.

2. Boevink, W., Kroon, H., van Vugt, M., Delespaul, P., & van Os, J. (2016). A user-developed, user run recovery programme for people with severe mental illness: A randomised control trial. Psychosis, 8, 287-300.

3. Castelein, S., Bruggeman, R., van Busschbach, J. T., van der Gaag, M., Stant, A. D., Knegtering, H., & Wiersma, D. (2008). The effectiveness of peer support groups in psychosis: A randomized controlled trial. Acta Psychiatrica Scandinavica, 118(1), 64-72.

4. Cook, J. A., Copeland, M. E., Floyd, C. B., Jonikas, J. A., Hamilton, M. M., Razzano, L., . . . Boyd, S. (2012a). A randomized controlled trial of effects of Wellness Recovery Action Planning on depression, anxiety, and recovery. Psychiatr Serv, 63(6), 541-547.

5. Cook, J. A., Steigman, P., Pickett, S., Diehl, S., Fox, A., Shipley, P., . . . Burke-Miller, J. K. (2012b). Randomized controlled trial of peer-led recovery education using Building Recovery of Individual Dreams and Goals through Education and Support (BRIDGES). Schizophr Res, 136(1), 36-42.

6. Corrigan, P., Sheehan, L., Morris, S., Larson, J. E., Torres, A., Lara, J. L., . . . Doing, S. (2018). The Impact of a Peer Navigator Program in Addressing the Health Needs of Latinos With Serious Mental Illness. Psychiatr Serv, 69(4), 456-461.

7. Corrigan, P. W., Kraus, D. J., Pickett, S. A., Schmidt, A., Stellon, E., Hantke, E., & Lara, J. L. (2017). Using Peer Navigators to Address the Integrated Health Care Needs of Homeless African Americans With Serious Mental Illness. Psychiatr Serv, 68(3), 264-270.

8. Craig T, Doherty I, Jamieson-Craig R, et al: The consumer-employee as a member of a Mental Health Assertive Outreach Team: I. clinical and social outcomes. Journal of Mental Health 13:59–69, 2004

9. Davidson, L., Shahar, G., Stayner, D. A., Chinman, M. J., Rakfeldt, J., & Tebes, J. K. (2004). Supported socialization for people with psychiatric disabilities: Lessons from a randomized controlled trial. Journal of Community Psychology, 32(4), 453-477.

10. Dennis, C. L. (2003). The effect of peer support on postpartum depression: a pilot randomized controlled trial. Can J Psychiatry, 48(2), 115-124.

11. Dennis, C. L., Hodnett, E., Kenton, L., Weston, J., Zupancic, J., Stewart, D. E., & Kiss, A. (2009). Effect of peer support on prevention of postnatal depression among high risk women: multisite randomised controlled trial. Bmj, 338, a3064.

12. Gjerdingen, D. K., McGovern, P., Pratt, R., Johnson, L., & Crow, S. (2013). Postpartum doula and peer telephone support for postpartum depression: a pilot randomized controlled trial. J Prim Care Community Health, 4(1), 36-43.

13. Griffiths, K. M., Mackinnon, A. J., Crisp, D. A., Christensen, H., Bennett, K., & Farrer, L. (2012). The effectiveness of an online support group for members of the community with depression: A randomised controlled trial. PLoS One, 7(12).

14. Johnson, S., Lamb, D., Marston, L., Osborn, D., Mason, O., Henderson, C., . . . Lloyd-Evans, B. (2018). Peer-supported self-management for people discharged from a mental health crisis team: a randomised controlled trial. Lancet, 392(10145), 409-418.

15. Kaplan, K., Salzer, M. S., Solomon, P., Brusilovskiy, E., & Cousounis, P. (2011). Internet peer support for individuals with psychiatric disabilities: A randomized controlled trial. Soc Sci Med, 72(1), 54-62.

16. Letourneau, N., Stewart, M., Dennis, C. L., Hegadoren, K., Duffett-Leger, L., & Watson, B. (2011). Effect of home-based peer support on maternal-infant interactions among women with postpartum depression: a randomized, controlled trial. Int J Ment Health Nurs, 20(5), 345-357.

17. Ludman, E. J., Simon, G. E., Grothaus, L. C., Luce, C., Markley, D. K., & Schaefer, J. (2007). A pilot study of telephone care management and structured disease self-management groups for chronic depression. Psychiatr Serv, 58(8), 1065-1072. ^a^

18. Mahlke, C. I., Priebe, S., Heumann, K., Daubmann, A., Wegscheider, K., & Bock, T. (2017). Effectiveness of one-to-one peer support for patients with severe mental illness-A randomised controlled trial. European Psychiatry, 42, 103-110.

19. O'Connell, M. J., Sledge, W. H., Staeheli, M., Sells, D., Costa, M., Wieland, M., & Davidson, L. (2018). Outcomes of a Peer Mentor Intervention for Persons With Recurrent Psychiatric Hospitalization. Psychiatr Serv, 69(7), 760-767.

20. Pfeiffer, P. N., King, C., Ilgen, M., Ganoczy, D., Clive, R., Garlick, J., . . . Valenstein, M. (2019). Development and pilot study of a suicide prevention intervention delivered by peer support specialists. Psychol Serv, 16(3), 360-371.

21. Ranzenhofer, L. M., Wilhelmy, M., Hochschild, A., Sanzone, K., Walsh, B. T., & Attia, E. (2020). Peer mentorship as an adjunct intervention for the treatment of eating disorders: A pilot randomized trial. International Journal of Eating Disorders, 53(5), 497-509.

22. Rivera, J. J., Sullivan, A. M., & Valenti, S. S. (2007). Adding consumer-providers to intensive case management: does it improve outcome?. Psychiatric Services, 58(6), 802-809.

22. Rogers, E. S., Maru, M., Johnson, G., Cohee, J., Hinkel, J., & Hashemi, L. (2016). A randomized trial of individual peer support for adults with psychiatric disabilities undergoing civil commitment. Psychiatr Rehabil J, 39(3), 248-255.

23. Rüsch, N., Abbruzzese, E., Hagedorn, E., Hartenhauer, D., Kaufmann, I., Curschellas, J., . . . Corrigan, P. W. (2014). Efficacy of Coming Out Proud to reduce stigma's impact among people with mental illness: pilot randomised controlled trial. Br J Psychiatry, 204(5), 391-397.

24. Russinova, Z., Rogers, E. S., Gagne, C., Bloch, P., Drake, K. M., & Mueser, K. T. (2014). A randomized controlled trial of a peer-run antistigma photovoice intervention. Psychiatr Serv, 65(2), 242-246.

25. Salzer, M. S., Rogers, J., Salandra, N., O'Callaghan, C., Fulton, F., Balletta, A. A., . . . Brusilovskiy, E. (2016). Effectiveness of peer-delivered Center for Independent Living supports for individuals with psychiatric disabilities: A randomized, controlled trial. Psychiatr Rehabil J, 39(3), 239-247.

26. Shorey, S., Chee, C. Y. I., Ng, E. D., Lau, Y., Dennis, C. L., & Chan, Y. H. (2019). Evaluation of a Technology-Based Peer-Support Intervention Program for Preventing Postnatal Depression (Part 1): Randomized Controlled Trial. J Med Internet Res, 21(8), e12410.

27. Solomon P, Draine J: The efficacy of a consumer case management team: 2-year outcomes of a randomized trial. Journal of Mental Health Administration 22:135–146, 1995

28. van Gestel-Timmermans, H., Brouwers, E. P., van Assen, M. A., & van Nieuwenhuizen, C. (2012). Effects of a peer-run course on recovery from serious mental illness: a randomized controlled trial. Psychiatr Serv, 63(1), 54-60.

List of studies narratively described, with a clinician-led intervention as a comparator condition (*n* = 3):

29. Field, T., Diego, M., Delgado, J., & Medina, L. (2013). Peer support and interpersonal psychotherapy groups experienced decreased prenatal depression, anxiety and cortisol. Early human development, 89(9), 621-624.

30. Mathews, C. A., Mackin, R. S., Chou, C. Y., Uhm, S. Y., Bain, L. D., Stark, S. J., ... & Delucchi, K. (2018). Randomised clinical trial of community-based peer-led and psychologist-led group treatment for hoarding disorder. BJPsych open, 4(4), 285-293.

31. Ludman, E. J., Simon, G. E., Grothaus, L. C., Luce, C., Markley, D. K., & Schaefer, J. (2007). A pilot study of telephone care management and structured disease self-management groups for chronic depression. Psychiatr Serv, 58(8), 1065-1072. ^a^

*^a^ The study from Ludman et al. (2007) is included in the meta-analysis as well as narratively described since the peer support intervention is compared to both an inactive control condition and a clinician-led control condition.*

**Appendix F**

**Narrative description of three RCTs with a clinician-led comparator**

Only three papers (Field et al., 2013; Ludman et al., 2007; Mathews et al., 2018) that met our inclusion criteria compared a Peer Support Intervention (PSI) to a clinician-led control condition and were, due to this limited number, not included in the meta-analysis. The three studies were conducted in the USA, examining PSIs with face-to-face delivery and group format. The studies included heterogeneous samples (see Appendix D).

Field et al. (2013) compared a 3-month unstructured PSI with an Interpersonal Psychotherapy (IPT) Group in 44 patients with a clinical diagnosis of prenatal depression, recruited from 2 medical centres. The study was rated as high risk of bias. A significant decrease of depression symptoms (measured by the Center for Epidemiologic Studies Depression [CES-D]) was reported in both the peer support condition and the psychotherapy control condition. The decrease was greater in the peer support group, though results should be interpreted with caution due to low power.

Ludman et al. (2007) compared an 1.5-month structured PSI with a professionally-led psychotherapy group with principles of Cognitive Behavioral Therapy (CBT) in 52 patients scoring above a cut-off level on a depression measure, which were recruited in a clinical setting. The risk of bias (RoB) for this study was rated as high risk. Although differences were not significant, 24% was diagnosed with a depressive disorder in the peer support condition at the end of treatment compared to 20% in the clinician-led control condition. Also, the Hopkins Symptom Checklist (HSCL) depression scores did not differ significantly between groups. The sample was too small to reliably detect differences in clinical outcomes.

The non-inferiority trial of Mathews et al. (2018) compared a 5-month unstructured PSI with a clinician-led group CBT in 323 individuals with hoarding disorder scoring above a cut-off level on a hoarding disorder symptom measure. Both inpatients, outpatients, and individuals in the general community were recruited for participation. The risk of bias for this study was rated as high risk. Mathews et al. (2018) reported a reduction of symptoms (assessed by the Saving Inventory-Revised [SI-R]) with an effect size of 1.20 for the peer-led group, and 1.21 for the clinician-led control condition, with no significant differences between them.

Overall, the quality of studies was low, with an overall score of high risk (see Figure G2 in Appendix G, and the table in Appendix H). Therefore, results should be considered with caution. Collectively, the effects of the interventions were primarily measured in terms of clinical recovery, with 2 trials (Field et al., 2013; Mathews et al., 2018) indicating that peer-led groups were as effective as psychologist-led groups for reducing symptom severity.

**References of RCTs with a clinician-led comparator**

1. Field T, Diego M, Delgado J, Medina L. Peer support and interpersonal psychotherapy groups experienced decreased prenatal depression, anxiety and cortisol. *Early Hum Dev.* 2013;89(9):621-624.
2. Ludman EJ, Simon GE, Grothaus LC, Luce C, Markley DK, Schaefer J. A pilot study of telephone care management and structured disease self-management groups for chronic depression. *Psychiatr Serv.* 2007;58(8):1065-1072.
3. Mathews CA, Mackin RS, Chou CY, et al. Randomised clinical trial of community-based peer-led and psychologist-led group treatment for hoarding disorder. *BJPsych Open.* 2018;4(4):285-293.

**Appendix G**

**Risk of Bias Graphs**

**Figure G1**

*Risk of Bias Graphs: Review Authors’ Judgments About Each Risk of Bias Item Presented as Percentages Across Included Studies in the Meta-Analysis (n = 28)*

**Figure G2**

*Risk of Bias Graph: Review Authors’ Judgments About Each Risk of Bias Item Presented as Percentages Across Included Studies narratively described (n = 3)*

**Appendix H**

**Risk of Bias rating per study: subdomains and overall rating using the Cochrane Collaboration Risk of Bias (RoB) tool 2.0**

| Author, year of publication | Domain 1  *The randomization process* | Domain 2  *Deviations from the intended interventions* | Domain 3  *Missing outcome data* | Domain 4 *^a^*  *Inappropriate measurement of the outcome* | Domain 5  *Selection of the reported results* | Overall RoB rating |
| --- | --- | --- | --- | --- | --- | --- |
| Studies included in the meta-analysis  (with CAU, WL or Other inactive control condition as comparator) | | | | | | |
| Boevink, 2016 | Some concerns | Low risk | Some concerns | Some concerns | Some concerns | High risk |
| Castelein, 2008 | Low risk | Low risk | Low risk | Some concerns | Some concerns | Some concerns |
| Cook, 2012a | Low risk | Low risk | High risk | Low risk | Some concerns | High risk |
| Cook, 2012b | Low risk | Low risk | High risk | Low risk | Some concerns | High risk |
| Corrigan, 2017 | Some concerns | Low risk | Low risk | Some concerns | Some concerns | Some concerns |
| Corrigan, 2018 | High risk | High risk | High risk | Some concerns | Some concerns | High risk |
| Craig, 2004 | Some concerns | Low risk | Low risk | Low risk & Some concerns | Some concerns | Some concerns |
| Davidson, 2004 | Some concerns | High risk | High risk | Some concerns | Some concerns | High risk |
| Dennis, 2003 | Low risk | Low risk | Low risk | Low risk | Some concerns | Low risk |
| Dennis, 2009 | Low risk | Low risk | Some concerns | Low risk | Some concerns | Some concerns |
| Gjerdingen, 2013 | High risk | Low risk | Low risk | Some concerns | Some concerns | High risk |
| Griffiths, 2012 | Low risk | Low risk | High risk | Some concerns | Low risk | High risk |
| Johnson, 2018 | High risk | Low risk | Some concerns | Low risk & Some concerns | Low risk | High risk |
| Kaplan, 2011 | Some concerns | Low risk | Low risk | Some concerns | Some concerns | Some concerns |
| Letourneau, 2011 | Some concerns | Low risk | High risk | Low risk | Some concerns | High risk |
| Ludman, 2007 | Some concerns | Low risk | High risk | Low risk | Some concerns | High risk |
| Mahlke, 2017 | Low risk | Low risk | High risk | Low risk & Some concerns | Some concerns | High risk |
| O'Connell, 2018 | Some concerns | Low risk | High risk | Low risk | Some concerns | High risk |
| Pfeiffer, 2019 | Some concerns | Low risk | High risk | Some concerns | Some concerns | High risk |
| Ranzenhofer, 2020 | Low risk | Low risk | Low risk | Some concerns | Low risk | Low risk |
|  |  |  |  |  |  |  |
|  |  |  |  |  |  |  |
| Author, year of publication | Domain 1  *The randomization process* | Domain 2  *Deviations from the intended interventions* | Domain 3  *Missing outcome data* | Domain 4 *^a^*  *Inappropriate measurement of the outcome* | Domain 5  *Selection of the reported results* | Overall RoB rating |
| Rivera, 2007 | Some concerns | High risk | Some concerns | Low risk | Some concerns | High risk |
| Rogers, 2016 | High risk | High risk | High risk | Some concerns | Some concerns | High risk |
| Rüsch, 2014 | Low risk | Low risk | High risk | Some concerns | Some concerns | High risk |
| Russinova, 2014 | Some concerns | Low risk | Low risk | Some concerns | Some concerns | Some concerns |
| Salzer, 2016 | Some concerns | Low risk | High risk | Some concerns | Some concerns | High risk |
| Shorey, 2019 | Low risk | Low risk | Low risk | Some concerns | Low risk | Low risk |
| Solomon, 1995 | High risk | Some concerns | Low risk | Low risk | Some concerns | High risk |
| van Gestel-Timmermans, 2012 | Low risk | Low risk | High risk | Some concerns | Some concerns | High risk |
| Studies narratively described in the systematic review  (with clinician-led control condition as comparator) | | | | | | |
| Field, 2013 | High risk | High risk | High risk | Some concerns | Some concerns | High risk |
| Ludman, 2007 | Some concerns | Low risk | High risk | Low risk | Some concerns | High risk |
| Mathews, 2018 | Low risk | High risk | High risk | Some concerns | Low risk | High risk |
| Abbreviations: CAU = Care-as-usual; RoB = Risk of Bias; WL = Waiting List. ^a^ Three trials were rated at both low risk and some concerns for bias in domain 4 due to the use of other types of measurement instruments per outcome category (e.g., using self-report measures for clinical recovery, and blinded-clinician rated instruments for personal recovery). | | | | | | |

**Appendix I**

**Subgroup analyses on moderators per outcome category: Clinical, Personal, and Functional Recovery Outcomes, Hedges *g***

|  | | Clinical Recovery | | |  | Personal Recovery | | |  | Functional Recovery | | |
| --- | --- | --- | --- | --- | --- | --- | --- | --- | --- | --- | --- | --- |
| Moderator | | No. of studies | *g* [95% CI] | *p* |  | No. of studies | *g* [95% CI] | *p* |  | No. of studies | *g* [95% CI] | *p* |
| Type of disorder | |  |  |  |  |  |  |  |  |  |  |  |
|  | Individuals with depressive symptoms ^a^ | 7 | 0.19 [-0.12, 0.51] | 0.95 |  | 2 | 0.18 [-0.02, 0.37] | 0.83 |  | 6 | 0.02 [-0.25, 0.29] | 0.65 |
|  | Serious Mental Illness (SMI) ^b^ | 14 | 0.18 [0.11, 0.25] |  |  | 17 | 0.15 [0.03, 0.27] |  |  | 18 | 0.08 [-0.02, 0.19] |  |
| Inclusion ^c^ | |  |  |  |  |  |  |  |  |  |  |  |
|  | Cut-off | 6 | 0.18 [-0.18, 0.55] | 0.93 |  | NA | NA |  |  | 6 | 0.02 [-0.25, 0.29] | 0.41 |
|  | Diagnosis | 13 | 0.20 [0.12, 0.27] |  |  | NA | NA |  |  | 15 | 0.14 [0.02, 0.26] |  |
| Recruitment | |  |  |  |  |  |  |  |  |  |  |  |
|  | Clinical | 13 | 0.17 [0.08, 0.26] | 0.64 |  | 11 | 0.11 [-0.07, 0.29] | 0.25 |  | 14 | 0.07 [-0.09, 0.22] | 0.76 |
|  | Other/mixed ^d^ | 9 | 0.21 [0.08, 0.34] |  |  | 8 | 0.22 [0.15, 0.29] |  |  | 11 | 0.09 [0.01, 0.19] |  |
| Delivery ^e^ | |  |  |  |  |  |  |  |  |  |  |  |
|  | In distance | 5 | 0.22 [0.10, 0.34] | 0.74 |  | 3 | 0.16 [0.06, 0.26] | 0.57 |  | 5 | 0.14 [0.01, 0.26] | 0.45 |
|  | In person | 13 | 0.16 [0.09, 0.24] |  |  | 15 | 0.12 [0.04, 0.21] |  |  | 15 | 0.04 [-0.04, 0.12] |  |
|  | Mixed | 4 | 0.20 [-0.31, 0.70] |  |  | NA | NA |  |  | 5 | 0.12 [-0.34, 0.58] |  |
| Format | |  |  |  |  |  |  |  |  |  |  |  |
|  | Group | 6 | 0.16 [0.07, 0.25] | 0.69 |  | 9 | 0.20 [0.10, 0.29] | 0.54 |  | 6 | 0.12 [0.08, 0.17] | 0.44 |
|  | Individual | 15 | 0.19 [0.09, 0.30] |  |  | 10 | 0.13 [-0.07, 0.33] |  |  | 18 | 0.06 [-0.08, 0.21] |  |
| Comparator | |  |  |  |  |  |  |  |  |  |  |  |
|  | CAU | 12 | 0.23 [0.15, 0.31] | 0.38 |  | 10 | 0.18 [-0.01, 0.36] | 0.33 |  | 15 | 0.05 [-0.11, 0.20] | 0.81 |
|  | Other active or inactive control | 4 | 0.14 [-0.06, 0.34] |  |  | 2 | -0.06 [-0.38, 0.26] |  |  | 3 | 0.17 [-0.19, 0.46] |  |
|  | WL | 6 | 0.09 [-0.14, 0.31] |  |  | 7 | 0.20 [0.09, 0.31] |  |  | 7 | 0.10 [0.00, 0.21] |  |
| Specific subgroup ^f^ | |  |  |  |  |  |  |  |  |  |  |  |
|  | Yes | 8 | 0.17 [-0.09, 0.42] | 0.87 |  | 6 | 0.09 [-0.05, 0.23] | 0.44 |  | 10 | -0.01 [-0.18, 0.15] | 0.18 |
|  | No | 14 | 0.19 [0.12, 0.26] |  |  | 13 | 0.17 [0.03, 0.31] |  |  | 15 | 0.12 [0.01, 0.24] |  |
| Perinatal depression | |  |  |  |  |  |  |  |  |  |  |  |
|  | Yes | 5 | 0.19 [-0.28, 0.66] | 0.99 |  | NA | NA |  |  | 20 | 0.10 [-0.01, 0.20] | 0.46 |
|  | No | 17 | 0.19 [0.12, 0.25] |  |  | NA | NA |  |  | 5 | -0.04 [-0.39, 0.31] |  |
| Structured vs unstructured ^g^ | |  |  |  |  |  |  |  |  |  |  |  |
|  | Structured | 7 | 0.11 [-0.02, 0.25] | 0.13 |  | 9 | 0.23 [0.05, 0.42] | 0.12 |  | 7 | 0.10 [-0.15, 0.36] | 0.80 |
|  | Unstructured | 14 | 0.24 [0.15, 0.32] |  |  | 10 | 0.06 [-0.04, 0.16] |  |  | 17 | 0.05 [-0.03, 0.17] |  |

*Note.* Abbreviations: CI = Confidence Interval; NA = Not Applicable
^a^ Participants scoring above a cut-off level on a depression scale for k = 6 (of which k = 5 are on perinatal depression); Participants with a clinical diagnosis of depression for k = 1.
^b^ A heterogeneous sample of clinically diagnosed mental health disorders, including diagnoses of depressive disorders for k=17, k=3 did not specify including depression.
^c^ Participants were included based on (1) scoring above a cut-off level on a validated measurement scale or (2) clinical diagnosis or documentation.
^d^ Studies with mixed recruitment included both a clinical group (inpatients and/or outpatients) and individuals recruited in the general population.
^e^ *In distance* refers to telephone and internet interventions; *In person* refers to face-to-face interventions; *Mixed* refers to a combination of different formats.
^f^ Whether the population is a specific subgroup of the general community (e.g., cultural background such as Latino’s) or aa patient subgroup (e.g., perinatal depression).
^g^ Whether the peer support intervention was structured (peer leaders followed a manual or pre-determined topics) or not (no manual, meetings based on mutual support solely).

**Appendix J**

**Funnel Plots**

**Figure J1**

*
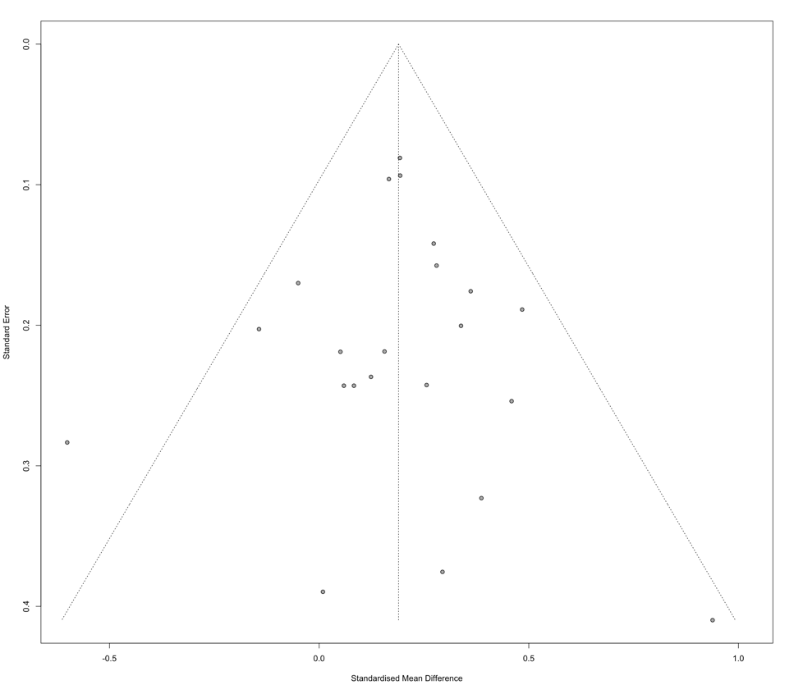
Funnel plot for clinical recovery*

*Note.* For clinical recovery, we found no indication that publication bias affected the results. Egger’s test of the asymmetry of the funnel plot was nonsignificant (*p* = 0.99). Adjusting for publication bias through the Duval and Tweedie’s trim and fill procedure showed an effect size of *g* = 0.18, 95% CI [0.10, 0.27], with one imputed study.

**Figure J2**

*
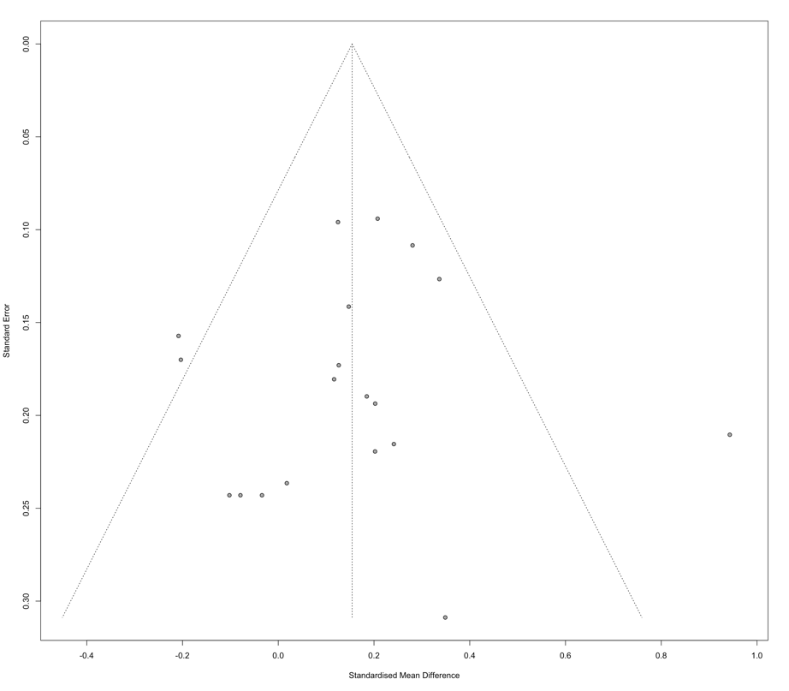
Funnel plot for personal recovery*

*Note.* For personal recovery we found no indication for publication bias, with Egger’s test nonsignificant (*p* = 0.66). Adjusting for publication bias through the Duval and Tweedie’s trim and fill procedure resulted in effect size *g* = 0.23, 95% CI [0.12, 0.35], with five imputed studies.

**Figure J3**

*
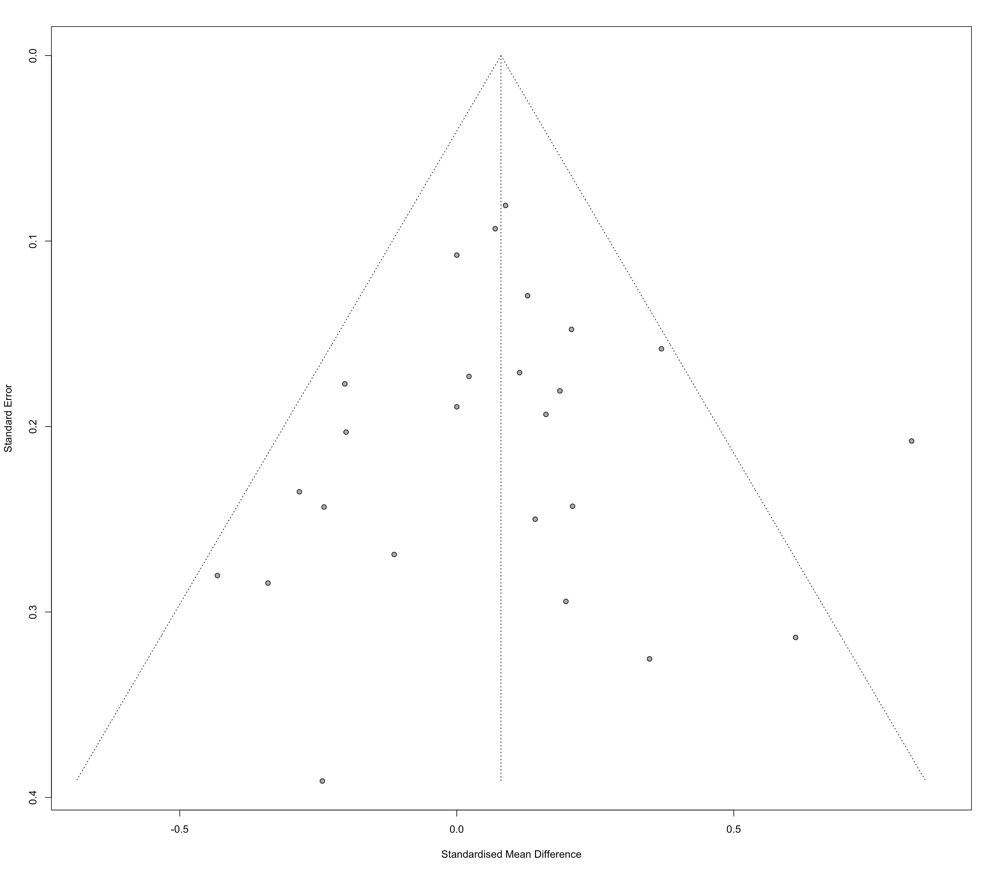
Funnel plot for functional recovery*

*Note.* For functional recovery we found no indication for publication bias, with Egger’s test nonsignificant (*p* = 0.74). Adjusting for publication bias through the Duval and Tweedie’s trim and fill procedure resulted in effect size *g* = 0.09, 95% CI [-0.01, 0.19], with one imputed study.

**Appendix K**

**Figure K1**

*Effect Sizes of Personal Recovery Outcomes*

*
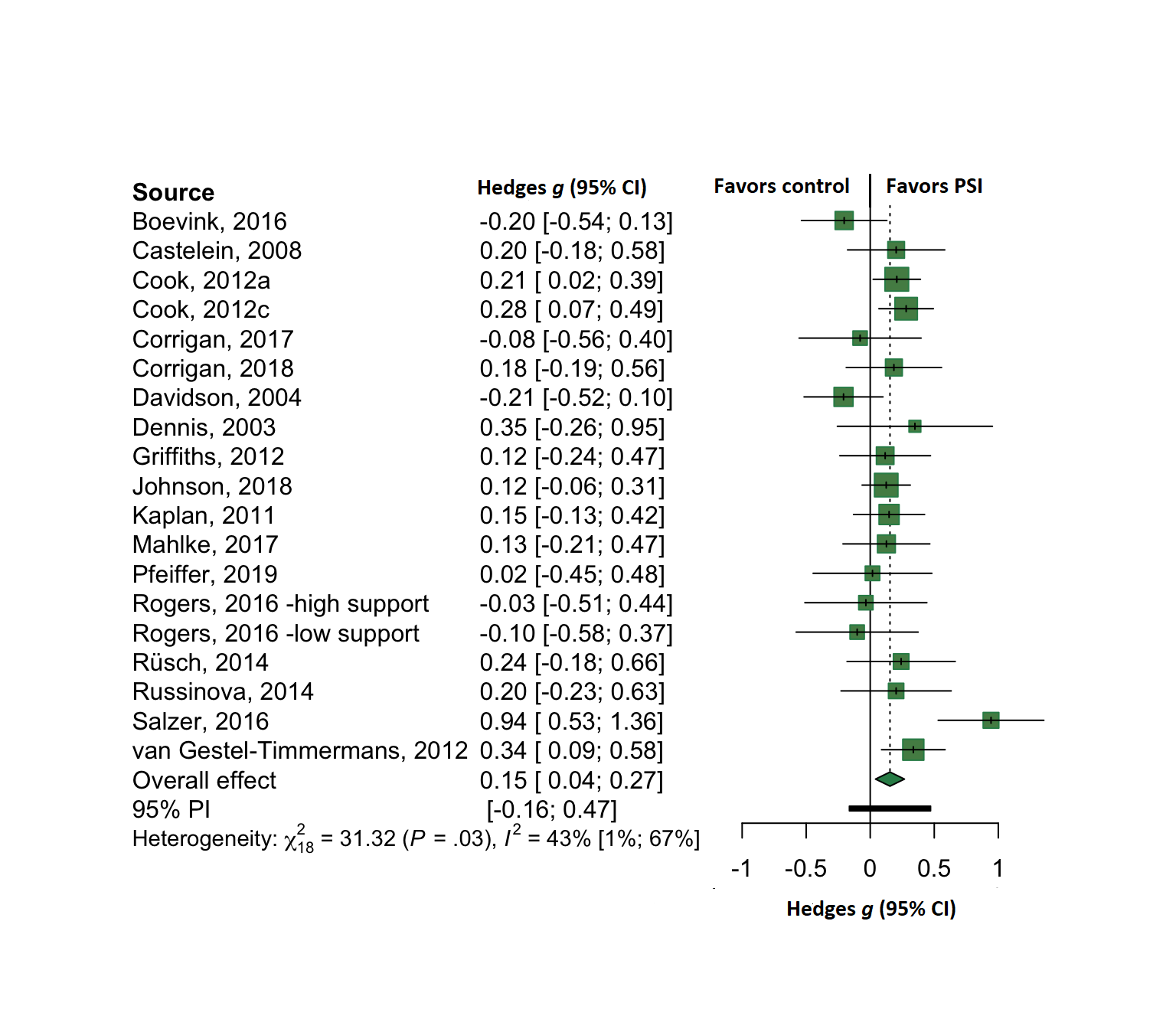
*

*Note.* Abbreviations: CI = Confidence Interval, PSI = Peer Support Intervention
Shown are standardized posttest effect sizes of comparisons between PSIs and control conditions for personal recovery relevant outcomes (Empowerment, Hope or overall personal recovery assessed by the Recovery Assessment Schedule [RAS]).

**Figure K2**

*Effect Sizes of Functional Recovery Outcomes*


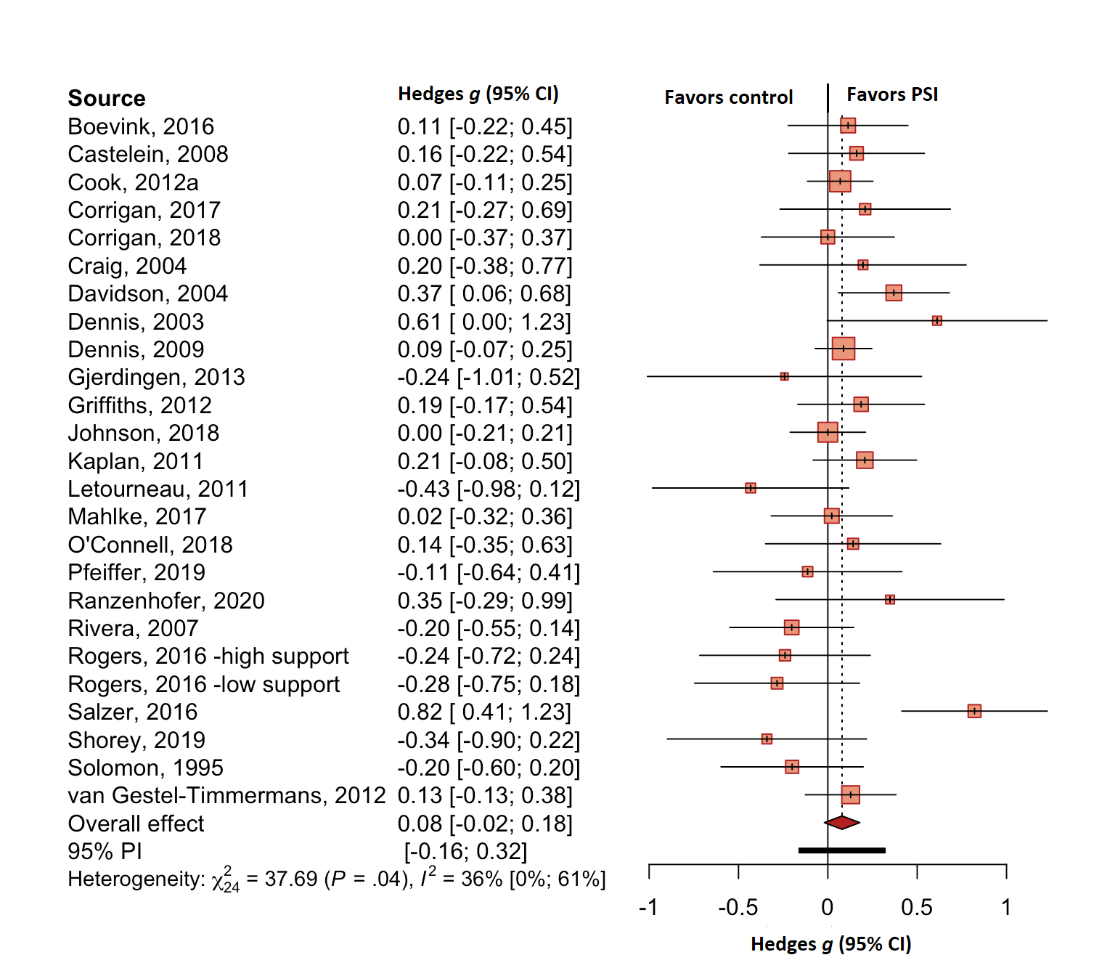


*Note.* Abbreviations: CI = Confidence Interval; PSI = Peer Support Intervention
Shown are standardized posttest effect sizes of comparisons between PSIs and control conditions for functional recovery relevant outcomes (i.e., Quality of Life or Social Functioning).
